# Self-supervised pretraining with NuSPIRe unlocks nuclear morphology-driven insights in spatial omics

**DOI:** 10.1186/s13059-026-03987-2

**Published:** 2026-02-03

**Authors:** Yuwei Hua, Shiyu Li, Yong Zhang

**Affiliations:** State Key Laboratory of Cardiovascular Diseases and Medical Innovation Center, Institute for Regenerative Medicine, Department of Neurosurgery, Shanghai Key Laboratory of Signaling and Disease Research, Frontier Science Center for Stem Cell Research, School of Life Sciences and Technology, Shanghai East Hospital, Tongji University, 1239 Siping Road, Shanghai, 200092 China

**Keywords:** Nuclear morphology, Self-supervised pretraining, Spatial transcriptomics, Representation learning

## Abstract

Nuclear morphology encodes rich phenotypic information critical for understanding cellular states, yet its full potential remains untapped in biomedical analysis. This study introduces NuSPIRe, a self-supervised deep learning model designed to analyze nuclear morphology using DAPI-stained images. Pretrained on 15.52 million cell nucleus images, NuSPIRe performs robustly in cell type identification and perturbation detection, even with limited annotations. Moreover, NuSPIRe integrates nuclear morphology with spatial omics data, uncovering significant correlations between cellular structure and gene expression. Notably, NuSPIRe further enables AI-driven experimental optimization for region-of-interest identification and field-of-view selection, enhancing data efficiency in spatial omics and molecular cell biology.

**Supplementary Information:**

The online version contains supplementary material available at 10.1186/s13059-026-03987-2.

## Background

DAPI (4',6-diamidino-2-phenylindole) staining is a widely used fluorescent dye that binds specifically to adenine–thymine (A-T) rich regions of DNA and emits strong blue fluorescence, delineating the positions and morphologies of cell nuclei [1]. This fluorescence staining technique, characterized by its operational simplicity, cost-effectiveness, and high specificity, is extensively employed across cellular biology, molecular biology, and medical research domains. The morphological features of DAPI staining, such as shape, brightness, and texture, are closely correlated with the DNA content and distribution within the cell nucleus [2]. These characteristics provide valuable insights for both quantitative and qualitative analyses, such as assessing cellular growth, divisional states, and potential pathological alterations [3–6]. Recent research has demonstrated that these morphological features hold substantial potential for further investigative and diagnostic development [7–9]. Furthermore, DAPI staining serves a vital role as an indicative marker during experiments. It helps researchers locate cellular positions and characterize the overall structure of tissues, guiding the identification of regions of interest during experimental setups. For instance, in fluorescence microscopy, DAPI staining can rapidly highlight regions with dense nuclei, enabling efficient localization of targets and precise focusing before data acquisition. Thus, DAPI staining serves a dual function—not only as a reliable tool for visualizing nuclear morphology but also as a strategic guide for optimizing experimental design and execution.

Despite being one of the most widely utilized tools for nuclear morphological analysis, existing methods for analyzing DAPI-stained images encounter significant challenges. Traditional approaches rely on manually crafted feature extraction techniques, such as edge detection, shape recognition, and texture analysis, which introduce subjectivity and inconsistencies in feature extraction [10]. Additionally, these methods struggle to handle subtle morphological variations and complex backgrounds, limiting their applicability in diverse conditions [11]. Although deep learning techniques have improved precision and reduced reliance on human-designed rules, they require extensively annotated datasets for effective supervised training [12, 13]. However, data annotation is time-consuming, resource-intensive, and particularly challenging in biomedical research involving rare diseases or specific biological samples [14, 15].

With the rapid advancement of spatial omics technologies, the challenges associated with utilizing DAPI staining as an indicator marker have become increasingly intricate. DAPI staining, a primary marker for spatial localization of tissues and cells, holds great potential for uncovering spatial heterogeneity at both single-cell and tissue levels. However, current experimental strategies fail to fully integrate the valuable information provided by DAPI staining into analytical frameworks and decision-making processes. In particular, the relationship between DAPI-derived morphological features and omics-level data, such as transcriptomics or proteomics, remains largely unexplored. This gap limits the integration of structural and molecular insights, hindering our ability to correlate spatial organization with functional biology. Moreover, experimental workflows often overlook the strategic utility of DAPI staining in guiding critical decisions, such as identifying regions of interest (ROI) or optimizing field-of-view (FOV) selection in complex tissue samples. These gaps highlight the urgent need for a more comprehensive incorporation of DAPI staining into analytical frameworks and experimental designs, bridging spatial visualization with omics-driven discovery.

To address these challenges, we developed the NuSPIRe, a novel deep learning model designed to extract and analyze morphological features from DAPI-stained images. NuSPIRe utilizes self-supervised pretraining on large-scale unannotated cell nucleus images, effectively extracting structural and morphological features of the nuclei without the need for extensive annotated datasets. NuSPIRe enhances the integration of morphological features with omics data, providing a critical link between structural characteristics and molecular insights. It supports a wide range of biomedical applications, including cell type identification and perturbation detection, even under challenging conditions with minimal sample availability or in the absence of annotations. Furthermore, NuSPIRe enhances the utility of DAPI staining by providing data-driven feedback that optimizes experimental workflows. This capability significantly reduces trial-and-error cycles, increases experimental efficiency, and improves overall success rates in research. By seamlessly integrating DAPI staining’s spatial insights with automated data analysis, NuSPIRe not only accelerates experimental data interpretation but also optimizes experimental workflows, directly advancing our understanding of cellular mechanisms and disease pathology.

## Results

### Overview of NuSPIRe architecture and training dataset

**Fig. 1:**
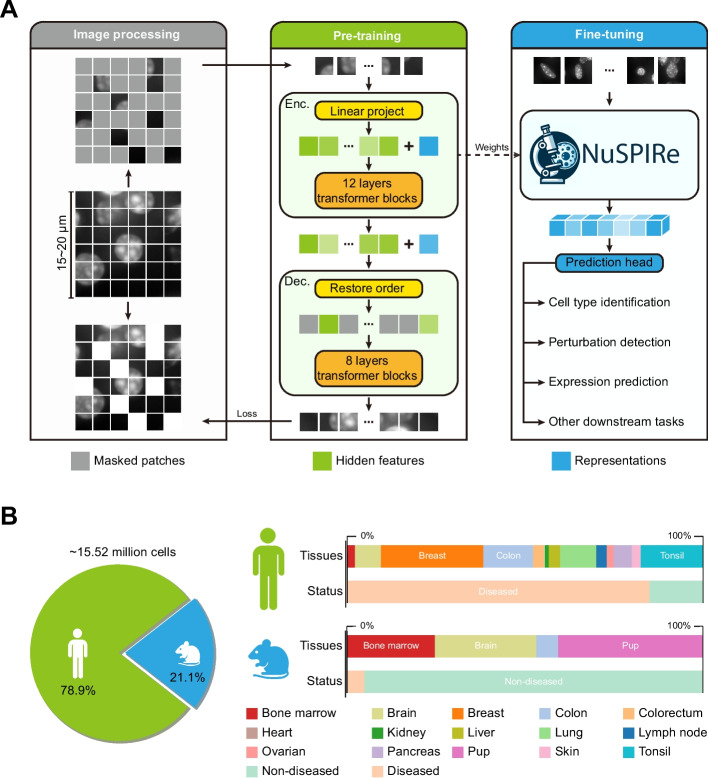
Overview of NuSPIRe Architecture and Training Dataset. **A** Schematic of the NuSPIRe model architecture. During the pretraining stage, DAPI-stained images are divided into non-overlapping patches, where a portion of patches are randomly masked. The encoder processes the unmasked patches to extract representations, which are passed to the decoder to reconstruct the masked patches. This self-supervised pretraining enables the model to learn global and local features of nuclear morphology. Fine-tuning is performed on smaller datasets for downstream tasks, including cell type identification, perturbation detection, and gene expression prediction. **B** Composition of the NuCorpus-15 M dataset used for pretraining. The dataset includes 15.52 million DAPI-stained cell nucleus images, 78.9% of which are derived from human tissues and 21.1% from mouse tissues. Samples span 15 organs or tissue types, including both diseased and non-diseased states, providing a diverse representation of nuclear morphology for model training

To extract morphological representations from DAPI-stained cell nucleus images, we adopted the pretraining-fine-tuning paradigm [16] commonly used in computer vision and developed a deep learning model named NuSPIRe. This model is based on self-supervised pretraining and involves two training stages: a general pretraining on a large-scale DAPI image dataset and a fine-tuning on smaller datasets tailored to specific downstream tasks (Fig. 1A, see Methods for details). During the pretraining stage, we adopted the Masked Image Modeling (MIM) [17] to train NuSPIRe in a self-supervised manner. Specifically, the input images are divided into non-overlapping patches, with most of these patches randomly masked. Only the unmasked patches are input into the NuSPIRe’s encoder to extract representations based on the visible parts of the image. The encoder’s output, along with the positional information of the masked patches, is passed to the decoder to reconstruct the masked content. The goal of this pretraining process is to enable the model to accurately reconstruct the masked patches, thereby learning both the global semantics and local details of the cell nucleus morphology. During the fine-tuning stage, the pretrained NuSPIRe’s encoder can be combined with different prediction heads to adapt to new datasets and specific downstream tasks, optimizing the model for various application scenarios.

To effectively pretrain NuSPIRe, we constructed a large-scale DAPI-stained cell nucleus image corpus named NuCorpus-15 M (Fig. 1B, see Methods for details). NuCorpus-15 M consists of 15.52 million high-quality cell nucleus images, of which 78.9% are derived from human tissues and 21.1% from mouse tissues. The dataset spans 15 different organs or tissue types, such as the breast, colon, brain, and tonsil, and broadly includes samples from both diseased and non-diseased states. These images were sourced from 56 standardized high-resolution tissue sections provided by the Xenium platform from 10 × Genomics, ensuring high consistency and reliability. The variety of tissue types and disease states in NuCorpus-15 M is crucial for enabling NuSPIRe to generalize across different biological contexts. The images in NuCorpus-15 M not only offer a substantial quantity but also provide a detailed depiction of subtle morphological differences in cell nuclei, including variations in nuclear size, shape, density, and arrangement. These variations are significant for cell biology research, as they allow NuSPIRe to learn to recognize critical morphological features. By pretraining on NuCorpus-15 M, the NuSPIRe learns to reconstruct nuclear images under various physiological and pathological conditions (Additional file 1: Fig. S1), gaining a robust understanding of nuclear morphology and establishing a valuable foundation for effectively tackling downstream tasks.

### Accurate cell type identification in the tumor immune microenvironment with limited annotated samples

**Fig. 2:**
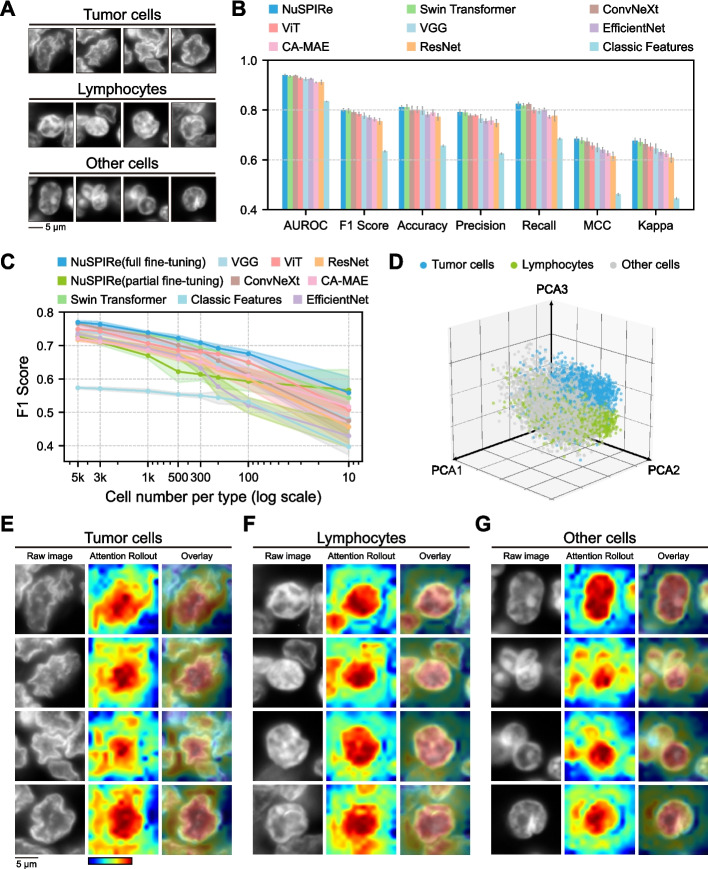
Accurate Cell Type Identification in the Tumor Immune Microenvironment with Limited Annotated Samples. **A** Representative DAPI-stained nuclear images of tumor cells, lymphocytes, and other cells from non-small cell lung cancer tissue sections. **B** Performance comparison of NuSPIRe and other models (VGG, ResNet, Swin Transformer, ConvNeXt, EfficientNet, ViT) across multiple metrics, including AUROC, F1 Score, Accuracy, Precision, Recall, MCC (Matthews Correlation Coefficient) and Cohen’s Kappa. Data represent mean values with standard deviations across five independent replications for each metric. **C** F1 Scores of different models across varying training sample sizes per cell type, displayed on a logarithmic scale. Shaded areas indicate the standard deviation around the mean values across five independent replications. **D** PCA plot illustrating the separation of tumor cells, lymphocytes, and other cells based on representations extracted by the pretrained NuSPIRe model. **E–G** Attention rollout visualizations for tumor cells **E**, lymphocytes **F**, and other cells **G**, highlighting the regions of the DAPI-stained images where the model focuses during classification

To validate NuSPIRe’s advantages in analysis tasks with limited annotated samples, we assessed its capability to accurately identify cell types in the tumor immune microenvironment. The accurate identification of cell types in clinical samples relies heavily on manual annotation, which often lacks standardized protocols [18]. We aimed to demonstrate that NuSPIRe can effectively distinguish tumor cells, lymphocytes, and other cells based on nuclear morphology in tissue samples where accurate cell type classification is essential for evaluating tumor immune status and guiding immunotherapy. We applied NuSPIRe to a DAPI-stained tissue section from a non-small cell lung cancer (NSCLC) sample, categorizing a total of 81,825 cells into three classes: tumor cells, lymphocytes, and other cells (Additional file 1: Fig. S2, see Methods for details). Previous studies have shown that tumor cell nuclei often exhibit abnormalities, such as enlarged nuclei, increased nucleocytoplasmic ratios, irregular nuclear membranes, and abnormal chromatin distribution [19]. By comparing the DAPI-stained images of the three cell types in the NSCLC tissue sections, we observed distinct differences in nuclear morphology between tumor cells and normal cells (Fig. 2A). We randomly extracted 5,000 nuclear images from each category as training data to fine-tune the pretrained NuSPIRe, and the results showed that NuSPIRe could effectively identify tumor cells and lymphocytes (Figs. 2B, S3, see Methods for details). Under full fine-tuning, we found that NuSPIRe outperformed other methods across various metrics (Fig. 2B). Notably, with partial fine-tuning (freezing the encoder and training only the prediction head), NuSPIRe retained most of this advantage, indicating a unique strength in nuclear morphology recognition (Additional file 1: Fig. S3). As expected, the performance of NuSPIRe trained from scratch without pretrained parameters showed inferior results, underscoring the necessity of extensive pretraining with large datasets to enhance its classification capabilities.

To verify the classification performance of NuSPIRe with minimal sample size, we gradually reduced the number of nuclear images used for fine-tuning (see Methods for details). The results indicated that, although the performance of all models deteriorated as training data was reduced, NuSPIRe consistently outperformed the alternatives (Fig. 2C). Notably, when the number of samples per category dropped below 300, other models exhibited a significant decline in performance. In contrast, NuSPIRe maintained performance comparable to other methods trained on 500 samples per category, even when only 10 samples per category were used. This highlights NuSPIRe’s effectiveness in scenarios with limited annotated data. Furthermore, the performance decline of NuSPIRe with partial fine-tuning was less pronounced than that of other methods. To explore the reasons behind our model’s robust performance, we utilized the pretrained NuSPIRe to extract representations from all cells. Remarkably, even without prior exposure to annotations, NuSPIRe was able to broadly distinguish between the three cell types based solely on the nuclear morphology representations extracted from DAPI-stained images (Fig. 2D). This demonstrates that the pretrained NuSPIRe can directly extract valuable morphological representations from unseen nuclear images.

To explore how NuSPIRe makes its classification decisions, we visualized the attention of the fine-tuned model using the Attention Rollout method [20], providing insights into its interpretability (see Methods for details). The attention maps revealed distinct patterns for each cell type (Fig. 2E-G). For tumor cells, the model mainly focused on the shape of the nucleus and the adjacent void area, suggesting that the shape and density of the nucleus are critical for identifying tumor cells (Fig. 2E). For lymphocytes, the model primarily focused on the shape of the nucleus and the shape of other nuclei around it, suggesting that the shape of the nucleus and its neighboring nuclei are important for identifying lymphocytes (Fig. 2F). For other cells, the model mainly focused on the dark regions within the nucleus, highlighting its emphasis on chromatin density and structural integrity of the nucleus (Fig. 2G). These results demonstrate the effectiveness of NuSPIRe in accurately identifying tumor and lymphocyte cells, particularly when annotated sample availability is limited, and underline its potential for enhancing clinical diagnostics.

### Annotation-free detection of perturbation-induced nuclear morphological responses

**Fig. 3:**
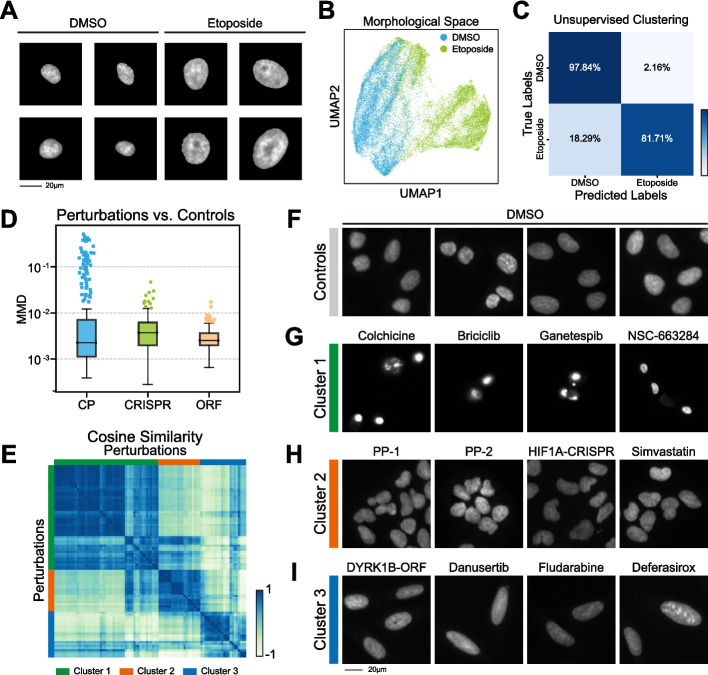
Annotation-free Detection of Perturbation-induced Nuclear Morphological Responses. **A** Representative DAPI-stained nuclear images of A549 cells treated with DMSO (control) and etoposide. **B** UMAP projection of nuclear morphological representations in DMSO- and etoposide-treated cells. **C** Clustering performance of Gaussian Mixture Method evaluated using Precision, Accuracy, Recall, and F1 Score. **D** Boxplot of Maximum Mean Discrepancy (MMD) between nuclear morphological representations of control cells and cells treated with different perturbations, including chemical compounds (CP), CRISPR knockouts (CRISPR), and overexpression of open reading frames (ORF). **E** Cosine similarity matrix comparing nuclear morphological responses across different significant perturbation conditions. **F-I** Representative DAPI-stained nuclear images showing three distinct nuclear clusters: **F** controls, **G** condensed chromatin, **H** abnormal nuclear morphology, and **I** nuclear enlargement and elongation

To verify NuSPIRe’s ability to handle analysis tasks in the absence of annotations, we applied it to detect drug-induced cellular senescence. Previous research has shown that senescent cells typically exhibit changes in chromatin structure and nuclear morphology [9, 21]. We utilized DAPI-stained images of treated A549 human lung adenocarcinoma cells, including samples treated with the chemotherapeutic agent etoposide (a topoisomerase II inhibitor) and dimethyl sulfoxide (DMSO) (Fig. 3A, see Methods for details). In this study, all cells treated with etoposide were assumed to be senescent, while those treated with DMSO were considered non-senescent [9]. We evaluated NuSPIRe’s ability to distinguish between these two cell types without additional training. We directly used the pretrained NuSPIRe to extract morphological representations from the nuclear images of all cells and found that these representations could effectively differentiate between senescent cells and control cells (Fig. 3B). Then, we employed unsupervised clustering methods to classify these cells using these representations (see Methods for details). The results demonstrated that the nuclear morphological representations extracted by NuSPIRe had significant discriminative power (Fig. 3C, Additional file 1: Fig. S4). Notably, even though NuSPIRe was not trained on any cell line DAPI-stained images during pretraining, it was still able to extract sufficient information to distinguish between these two cell types. This highlights NuSPIRe’s potential to detect drug-induced cellular changes without annotations.

To further test this potential, we attempted to characterize nuclear morphological changes under different types of perturbations using NuSPIRe without annotations. We utilized the resource dataset CPJUMP1 [22], which includes DAPI-stained nuclear images of cells treated with various paired chemical and genetic perturbations. From this dataset, we extracted nuclear morphological representations from U2OS cells treated with 303 compound treatments (CPs), 161 CRISPR-Cas9 knockouts (CRISPRs), and 176 open reading frame overexpressions (ORFs), as well as from negative control cells. To evaluate the impact of these perturbations on nuclear morphology, we calculated the Maximum Mean Discrepancy (MMD) between the perturbation-treated cells and the negative controls (see Methods for details). The results indicated that most treated cells exhibited minimal morphological divergence, suggesting that not all perturbations directly affect nuclear morphology (Fig. 3D). Consequently, we used the interquartile range (IQR) to identify perturbations that induced significant alterations in nuclear morphology (Additional file 1: Fig. S5, Additional file 2: Table S1, see Methods for details). Ultimately, we identified 92 distinct significant perturbations, including 67 CPs, 11 CRISPRs, and 14 ORFs, implying that chemical compound perturbations are more likely to induce a cellular nuclear response than gene-targeting perturbations. Then, we defined a vector in the morphological space extending from the negative control to each perturbation, representing the induced morphological change. By calculating the cosine similarity between these morphological vectors, we found that cells with significant nuclear changes could be broadly classified into three distinct clusters (Fig. 3E-I, Additional file 1: Fig. S6, Additional file 2: Table S2, see Methods for details). The first cluster, mainly induced by compounds such as colchicine, briciclib, ganetespib, and NSC-663284, is characterized by highly condensed chromatin (Fig. 3G). This interpretation is consistent with previous several studies reporting that these compounds can trigger cell cycle arrest or apoptotic responses in tumor cells [23–27]. The second cluster is marked by abnormal nuclear morphology, often implying aberrant cell division, with representative perturbations including PP-1, PP-2, simvastatin, and HIF1A-CRISPR (Fig. 3H). Previous reports have shown that these perturbations disrupt metabolic pathways that are essential for proper mitosis, supporting our observation [28–31]. The third cluster features nuclear enlargement and elongation, potentially indicating cellular senescence or DNA damage. Such nuclear changes have been observed following perturbations such as DYRK1B-ORF, danusertib, fludarabine and deferasirox (Fig. 3I). This pattern has been consistently reported across several studies in tumor cells [32–34]. When comparing nuclear morphological responses triggered by different perturbations targeting the same gene, we found that although it is challenging to accurately match perturbations across modalities based solely on the simple nuclear morphology clusters, NuSPIRe successfully identified several matched perturbations. For example, PP-2 and ABL1-ORF (cosine similarity = 0.83), AVL-292 and BTK-CRISPR (cosine similarity = 0.70), and SB-203580 and TNF-ORF (cosine similarity = 0.68) exhibited significant alignment. These findings are consistent with previous research [22], thereby demonstrating the potential of NuSPIRe to advance biomedical research and clinical applications, especially in drug screening. We further compared NuSPIRe with classical morphological features derived from all Cell Painting channels in the CPJUMP1 dataset (Additional file 1: Fig. S7). The analysis revealed a substantial overlap between perturbations identified by the two approaches, with 89.4% of those detected using Cell Painting also captured by NuSPIRe, indicating that nuclear morphology alone can recover most biologically meaningful responses. These findings suggest that NuSPIRe provides a lightweight and annotation-free alternative for perturbation screening when only nuclear stains are available.

### Gene expression prediction in the context of spatial omics

**Fig. 4:**
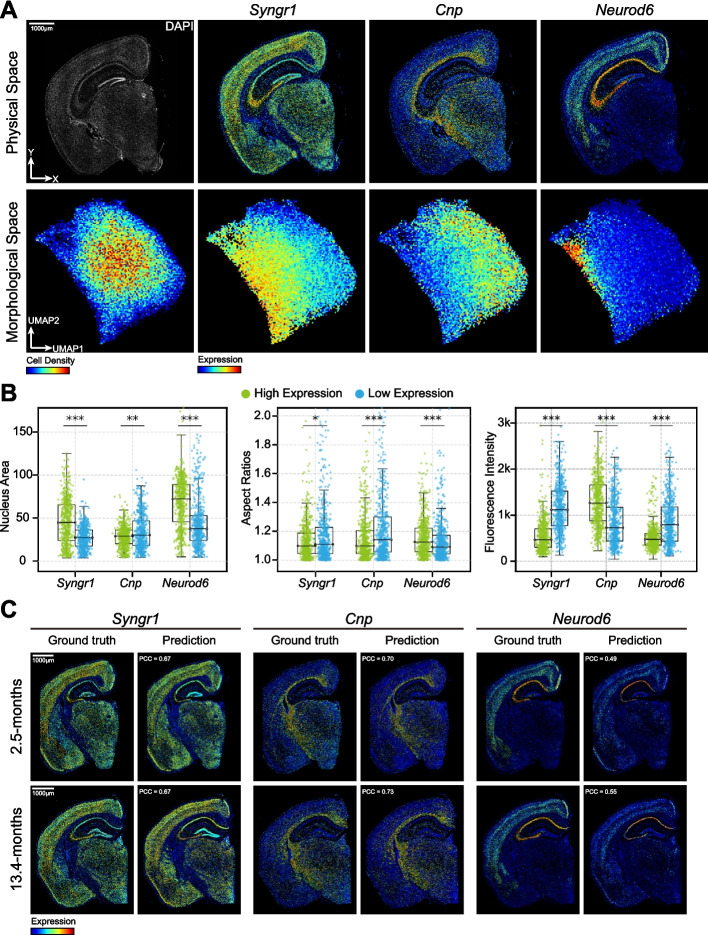
Gene Expression Prediction in the Context of Spatial Omics. **A** Spatial distribution of gene expression (*Syngr1*, *Cnp*, *Neurod6*) in the mouse brain at 5.7 months. Upper row: physical space showing DAPI-stained images with individual cells plotted at their spatial coordinates and colored by their gene expression levels. Lower row: UMAP projection of cells in morphological space using hexbin aggregation, where each hexagon summarizes multiple cells, and the color intensity reflects either the local cell density or the average gene expression level within that region. **B** Boxplots showing differences in the nuclear area, aspect ratio, and fluorescence intensity between high and low expression groups for *Syngr1*, *Cnp*, and *Neurod6*. **C** Ground truth and predicted gene expression maps for *Syngr1*, *Cnp*, and *Neurod6* at two time points (2.5-months and 13.4-months) in mouse brain. Pearson correlation coefficients (PCC) between predicted and actual gene expression values are indicated for each time point

To deeply explore NuSPIRe’s capability to leverage the potential of DAPI-stained histological images in spatial omics technologies, we analyzed spatial transcriptome data from a 5.7-months wild-type mouse brain tissue section. This dataset included gene expression profiles for 53,388 cells across 347 genes, along with a corresponding DAPI-stained image. The function and behavior of cells are directly regulated by specific gene expressions, often inducing subtle changes in nuclear morphology [35]. Consequently, the size, shape, and structure of cell nuclei can significantly reflect the active state of specific genes, providing insights into complex molecular mechanisms within cells. Using the pretrained NuSPIRe model, we extracted nuclear morphological representations from all cells and observed distinct distribution patterns in morphological space for genes such as *Syngr1*, *Cnp*, and *Neurod6*, confirming a significant correlation between nuclear morphology and gene expression (Fig. 4A, Additional file 1: Fig. S8).

To further investigate the correspondence between nuclear morphological features and gene expression, we fine-tuned the NuSPIRe model for the task of predicting gene expression profiles based on nuclear morphology. The fine-tuned NuSPIRe was capable of predicting gene expression profiles from nuclear morphology, outperforming other common methods (Additional file 1: Fig. S9, see Methods for details). This suggests that NuSPIRe has learned the relationships between specific gene expression levels and nuclear morphology during training. By evaluating the model’s prediction performance for each gene, we identified several genes, including *Syngr1*, *Cnp*, and *Neurod6*, whose expression is associated with nuclear morphology. Specifically, we found that the expression of these genes is strongly correlated with common morphological features such as nucleus area, aspect ratio, fluorescence intensity, and texture. To further validate these results, we analyzed the common morphological features of cells with both high and low expression levels of these genes, revealing significant differences between the two groups (Fig. 4B, Additional file 1: Fig. S10, see Methods for details). These findings suggest a strong link between the expression of these genes and nuclear morphology, providing valuable insights into cellular structure and function.

To validate the general applicability of the relationships learned by the NuSPIRe model, we examined spatial transcriptome data from two additional time points in mouse brains: 2.5-months and 13.4-months of age. Notably, the distribution of nuclear morphologies remained relatively consistent across the three time points (Additional file 1: Fig. S11). This consistency enabled us to apply the fine-tuned NuSPIRe model, originally trained on the 5.7-months data, directly to the datasets from the earlier and later time points. The model exhibited robust predictive performance across all samples, suggesting that the relationships it learned are broadly applicable rather than limited to a specific tissue section (Fig. 4C, Additional file 1: Fig. S12). This finding highlights the model’s versatility in diverse biological contexts, showcasing its potential to improve the efficiency and cost-effectiveness of spatial omics.

### ROI identification and FOV optimization in spatial omics experimental design

To fully explore the potential of NuSPIRe in guiding spatial omics workflows using DAPI staining, we aimed to leverage NuSPIRe to identify regions of interest (ROIs) based on DAPI-stained images, enabling cost-effective field of view (FOV) selection. During the pre-scanning process in spatial omics technologies, DAPI staining is commonly used as an efficient method for locating cells and tissues, providing crucial guidance for selecting FOVs. Traditional methods typically rely on dense sampling and stitching multiple FOVs to cover the ROI as comprehensively as possible [36–38]. However, increasing the number of FOVs directly leads to a substantial rise in reagent cost and resource consumption. Without targeted strategies, collecting data across entire tissue slices may waste considerable experimental cost on irrelevant regions. By harnessing NuSPIRe’s morphological analysis capabilities, we sought to focus on ROI identification in pre-scanned DAPI images, aiming to optimize FOV selection by targeting the most informative regions, improve experimental efficiency, and reduce unnecessary reagent use, thereby maximizing biological yield per unit cost.

**Fig. 5:**
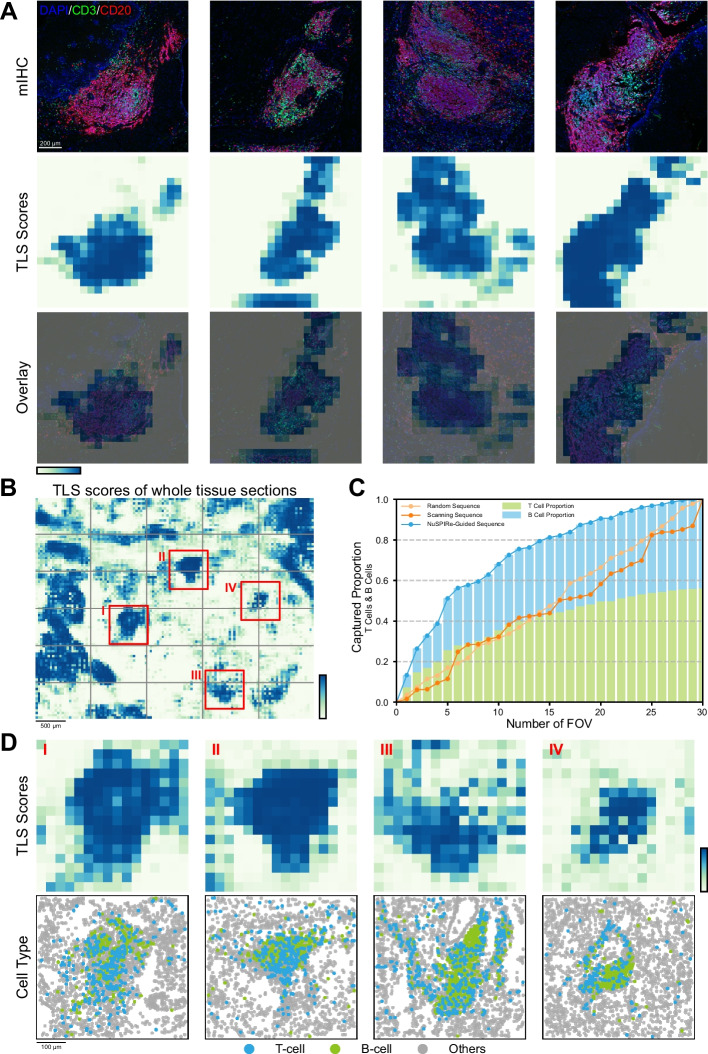
ROI Identification and FOV Optimization in Spatial Omics Experimental Design **A** The results of TLS identification using the fine-tuned NuSPIRe model on mIHC images. The top row shows mIHC images with DAPI (blue), CD3 (green), and CD20 (red) staining from ESCC tissue slices. The middle row presents TLS scores predicted by the NuSPIRe model, highlighting regions identified as TLS. The bottom row overlays the TLS scores onto the original mIHC images, demonstrating high concordance between TLS regions identified by NuSPIRe and the gold standard CD3 and CD20 staining. **B** Heatmap showing TLS scores predicted by the NuSPIRe model on a pre-scanned DAPI-stained image of the whole tissue section from NSCLC spatial transcriptomics data. Red boxes indicate four representative FOVs (I-IV) optimized by NuSPIRe. **C** Comparison of three FOV selection strategies: NuSPIRe-guided sequence, scanning sequence, and random sequence. The NuSPIRe-guided sequence captured a higher proportion of T cells and B cells within the top-ranked FOVs compared to other strategies. **D** The top row displays TLS score heatmaps for the four representative FOVs (I-IV) selected and optimized by the NuSPIRe model. High TLS score regions are highlighted, corresponding to potential TLS areas. The bottom row shows the cell type composition within these FOVs, with T cells, B cells, and other cells mapped in spatial distributions

To achieve this goal, we utilized NuSPIRe to recognize tertiary lymphoid structures (TLS), an important feature in the tumor immune microenvironment, based on cell nucleus morphology in tissue slices. Specifically, we employed a multi-immunohistochemistry (mIHC) image dataset derived from tissue samples of 64 esophageal squamous cell carcinoma (ESCC) patients, which included DAPI, CD3, and CD20 staining information [39]. By combining mIHC markers, we precisely identified TLS and non-TLS regions as ground truth for fine-tuning the NuSPIRe model (see Methods for details). Experimental results showed that the fine-tuned NuSPIRe could accurately identify TLS regions based on DAPI-stained histological images, demonstrating high consistency with the gold standard of CD3 and CD20 staining (Fig. 5A, Additional file 1: Fig. S13). This highlights that extracting morphological features from DAPI images not only enables effective identification of functional tissue regions but also reduces the reliance on multiplex staining, offering greater flexibility in experimental design.

We then simulated a practical application of NuSPIRe in FOV selection using a non-small cell lung cancer (NSCLC) spatial transcriptomics dataset, thereby illustrating its real-world impact. Specifically, we used pre-scanned DAPI-stained images from the experimental setup as references and employed the fine-tuned NuSPIRe model to identify TLS regions across the entire tissue slice, ranking each region by its acquisition value (Fig. 5B). Analyzing 30 densely sampled FOVs obtained under real experimental conditions, we found that selecting only the top 5 FOVs with the highest TLS scores captured more than half of the T cells and B cells, while selecting the top 15 FOVs covered over 80% of these lymphocytes (Fig. 5C). By comparison, FOV selection based on random sequence or scanning sequence resulted in markedly lower efficiency (Fig. 5C). These findings demonstrate NuSPIRe can substantially reduce the number of FOVs while ensuring comprehensive coverage of major ROIs. Additionally, we further analyzed the acquisition results and identified the potential to redesign FOV size and positioning based on NuSPIRe. By centering FOV layouts on ROIs, NuSPIRe enables more efficient capture of key tissue structures, improving both data quality and resolution. For example, four representative FOVs initially located at the edges of TLS regions were repositioned to center these structures (Fig. 5B, D), ensuring comprehensive data acquisition and minimizing the loss of critical structural information.

To further evaluate NuSPIRe beyond TLS detection, we tested its ability to predict immune infiltration levels directly from DAPI-stained images. Using weak labels derived from spatial transcriptomics, the predicted infiltration scores showed strong correlations with ground-truth immune cell densities across independent NSCLC sections (Additional file 1: Fig. S14, see Methods for details), indicating that NuSPIRe can capture graded and spatially heterogeneous immune infiltration patterns beyond discrete structures such as TLS.

These results validate the significant potential of NuSPIRe for guiding spatial omics workflows using DAPI-stained images, offering a new perspective on experimental design. This robust and flexible approach highlights NuSPIRe’s capacity to accelerate spatial omics studies, paving the way for more targeted and cost-effective data acquisition in diverse biomedical settings.

## Discussion

In this study, we introduced NuSPIRe, a self-supervised deep learning model for analyzing nuclear morphology using DAPI-stained images. Our findings show that NuSPIRe effectively extracts and analyzes nuclear morphological features, demonstrating significant improvements over traditional methods, particularly under data-limited conditions. For instance, NuSPIRe achieved higher accuracy in distinguishing tumor cells from lymphocytes compared to conventional techniques when only a small number of annotated samples were available. The model’s ability to generalize across different datasets and accurately identify cell types based on nuclear morphology highlights its potential for clinical applications, such as tumor microenvironment analysis and immune profiling. Furthermore, NuSPIRe can detect cellular changes and perturbations without requiring annotated data, making it an invaluable tool for drug screening. By predicting gene expression profiles from nuclear morphology, NuSPIRe provides a novel approach to linking nuclear structure with gene activity. Importantly, NuSPIRe optimizes spatial omics workflows by improving ROI identification and FOV selection, thus largely reducing data acquisition time and resource usage while maintaining comprehensive coverage.

While NuSPIRe demonstrates remarkable performance, DAPI staining primarily reflects nuclear and chromatin organization and therefore provides only a partial view of cellular state. In an exploratory cell-cycle staging analysis, the concordance between DAPI nuclear morphology and transcriptome-based cell-cycle annotations was limited (data not shown). This indicates a technical limitation and suggests a biological boundary for DAPI-only approaches to resolve subtle, fine-grained cellular states. To address this, integrating additional staining techniques or multi-modal imaging could enrich the representation beyond nuclear morphology. In parallel, more fine-grained and better-curated labeling strategies may improve robustness and extend NuSPIRe’s applicability.

In terms of generalization, highly heterogeneous tissues remain a key challenge. Although cross-sample prediction performs well in mouse brain, performance can degrade in more complex samples. In our tumor cell identification analysis, NuSPIRe achieves good results after lightweight fine-tuning across sections and patients, yet we observed a performance drop in a zero-shot setting. A plausible explanation is that the pretraining data do not yet span sufficiently broad variability in nuclear shape, size, and texture, acquisition settings, and microenvironmental effects. Nonetheless, lightweight fine-tuning provides a practical and effective strategy when transferring NuSPIRe to new scenarios. As a more fundamental solution, pretraining on a much expanded dataset covering multiple tissues, species, pathological contexts, and imaging platforms, such as Xenium, CosMx SMI, MERFISH, CODEX, and CyCIF, should better capture real-world variability, improve out-of-the-box robustness, and reduce reliance on platform-specific cues.

Another issue is the interpretability of NuSPIRe. While attention maps offer preliminary clues to the morphological cues used for classification, a mechanistic bridge from morphology to function remains incomplete. Although morphology-to-transcriptome correlations have been reported previously [40, 41], it remains challenging to determine which biological programs are truly captured by nuclear morphology. Two non-mutually exclusive mechanisms likely contribute. First, for genes encoding direct effectors of nuclear architecture (such as lamina components, heterochromatin regulators), changes in expression or activity can alter lamina tethering, chromatin compaction, and nuclear rigidity, leading to measurable nuclear morphology differences [42, 43]. In the mouse brain tissue dataset (347 genes assayed) used in this study, only *Satb2* and *Hat1* encode direct chromatin architecture effectors, and both showed significant links between their expression and nuclear morphology (Additional file 1: Fig. S10), consistent with previous reports [44, 45]. Second, since cell differentiation processes or physiological processes can simultaneously shape transcriptional programs and nuclear organization, expression of lineage-marker or state-associated genes can correlate with nuclear size or texture without directly determining morphology. In our mouse brain analysis, most top-associated genes are lineage-marker genes (for example, *Syngr1* and *Neurod6* for neuronal lineage, *Cnp* for oligodendrocyte lineage). Moving beyond descriptive associations, future works will prioritize explainability by coupling NuSPIRe with orthogonal assays and causal perturbations to delineate how nuclear morphology encodes regulatory mechanisms.

Methodologically, although NuSPIRe has demonstrated strong effectiveness, it still leaves substantial design space unexplored. NuSPIRe’s performance likely arises from the interplay among domain-specific pretraining, model architecture, and self-supervised strategy, highlighting the need for a more systematic investigation of their compatibility. Alternative self-supervised strategies such as SimCLR [46] and BYOL [47] remain promising for nuclear morphology representation learning and future model design will focus on further increasing representation capacity.

Looking forward, as spatial and molecular datasets become more comprehensive, NuSPIRe could be extended to an expanding range of biological tasks. These may include inferring proliferative and cell-cycle states, estimating immune infiltration, and characterizing other dynamic processes such as differentiation, senescence, or stress responses directly from nuclear morphology. Such extensions would further demonstrate the model’s ability to link morphological variation with diverse functional phenotypes across tissues and diseases.

## Conclusions

NuSPIRe is an intelligent and versatile framework that advances our understanding of cellular morphology and its relationship to gene expression. By integrating nuclear morphology with omics data, NuSPIRe offers a novel approach to functional genomics and biomarker discovery. NuSPIRe’s capacity to streamline spatial omics experiments by intelligently identifying ROIs and optimizing FOV selection underscores its potential to significantly reduce time and resource consumption, thereby accelerating research and clinical workflows. Future improvements in multi-modal data integration and model interpretability will further enhance NuSPIRe’s applicability, positioning it as a transformative system for computational biology and translational medicine.

## Methods

### Collection of the NuCorpus-15 M

We constructed a large-scale DAPI-stained cell nucleus image dataset, named NuCorpus-15 M, comprising 15.52 million high-quality images. These images were sourced from 56 standardized high-resolution tissue sections, provided by the Xenium platform from 10 × Genomics. For each high-resolution DAPI-stained image, we extracted sub-images centered on the geometric center of each cell nucleus, with a sub-image size of 20 µm on each side. To ensure the quality of the dataset, we applied fluorescence intensity thresholds to each high-resolution image, selectively screening the extracted sub-images. This step was crucial to remove sub-images containing abnormally stained nuclei. After screening, the remaining 15.52 million high-quality cell nucleus images were retained. To address variations in fluorescence intensity caused by imaging conditions, the intensity of each sub-image was normalized. This normalization step ensured consistency across all images, minimizing the impact of differences in staining or imaging equipment. All sub-images, along with their corresponding metadata, were stored in HDF5 format to enable efficient access and retrieval.

### NuSPIRe architecture

The NuSPIRe architecture is designed to learn effective representations by applying masked image modeling, where portions of the input image are masked and the model predicts the original signals for the masked regions. The architecture consists of the following key components:

#### Image patch division

The input image $X$ of size $H\times W$ is divided into $N$ non-overlapping patches, each of size $P\times P$. Each patch ${x}_{i}$ can be flattened into a vector, allowing the image to be represented as:

$$X=\left\{{x}_{1}\right.,{x}_{2},\cdots ,\left.{x}_{N}\right\}, N=\frac{H\times W}{P\times P}, {x}_{i}\in {\mathbb{R}}^{P\times P}$$

#### Linear projecting and positional encoding

Each flattened image patch ${x}_{i}$ is linearly projected into a vector of fixed dimension $d$ using a weight matrix ${W}_{p}$:

$${z}_{i}={W}_{p}{x}_{i}+{b}_{p}, {W}_{p}\in {\mathbb{R}}^{d\times \left(P\times P\right)}, {z}_{i}\in {\mathbb{R}}^{d}$$

And a representation token ${z}_{rep}\in {\mathbb{R}}^{d}$ ​is introduced to capture global image information about the entire image. Positional encodings ${E}_{pos}$​ are then added to both the representation token and the patch embeddings to maintain positional awareness:

$$Z=\left[{z}_{rep};{z}_{1};{z}_{2};\cdots ;{z}_{N}\right]+ {E}_{pos}$$

#### Masking

Next, we apply a random masking operation to the image patches. The mask matrix $M$ is a binary vector where each element ${M}_{i}$ indicates whether the corresponding image patch ${x}_{i}$ is masked (1 for masked, 0 for unmasked). Using the mask matrix $M$, we can define the observed (unmasked) image patches as:

$${X}_{obs}=X\odot \left(1-M\right)$$

Here, $\odot$ represents element-wise multiplication. ${X}_{obs}$​ contains all the unmasked patches, which are then input to the encoder along with the representation token:

$${Z}_{enc}=\left[{z}_{rep};{z}_{{obs}_{1}};{z}_{{obs}_{2}};\cdots ;{z}_{{obs}_{m}}\right]$$

where $m$ is the number of unmasked patches, and ${obs}_{1};{obs}_{2};\cdots ;{obs}_{m}$ are the indices of the unmasked patches.

#### Encoder

The encoder takes the unmasked image patches and the representation token as input. It is composed of $L$ stacked transformer blocks such that the output of one block is in the input of the following block. The output of the stacked transformer blocks can be defined as follows:

$${H}^{0}={Z}_{enc}$$

$${H}^{l}=\left[{h}_{rep}^{l};{h}_{1}^{l};{h}_{2}^{l};\cdots ;{h}_{m}^{l}\right]$$

$${H}^{l+1}={Transformer\_block}_{l}\left({H}^{l}\right), \forall l\in \left[0,L-1\right]$$

Each transformer block consists of two main modules: a multi-head self-attention mechanism and a feed-forward neural network [48]. The multi-head self-attention mechanism enables the model to weigh the relevance of different input elements in the input set when learning global representations. In our case, we use 12 stacked transformer blocks of 12 attention heads as the encoder of NuSPIRe. The outputs of encoder can be defined as follows:

$$H=Encoder\left({Z}_{enc}\right)$$

After pretraining, the representation token ${h}_{rep}$ will be treated as a global representation of the entire image.

#### Decoder

The decoder’s function is to reconstruct the original image, including the masked patches. Before the decoder can operate, the representation token ${h}_{rep}$ is removed from $H$:

$${H}_{obs}=\left[{h}_{1};{h}_{2};\cdots ;{h}_{m}\right]$$

This leaves only the encoded features of the unmasked patches. To reconstruct the original image, the mask tokens ${h}_{mask}$ are added back into ${H}_{obs}$ at the positions corresponding to the masked patches. Each mask token is a learnable vector of the same dimensionality as ${h}_{i}$:

$${H}_{all}=Insert\left({h}_{mask},{H}_{obs}\right)$$

Here, the function $Insert()$ denotes the process of inserting the mask tokens at their corresponding positions, resulting in a sequence that matches the original length $H$ of the input image patches:

$${H}_{all}=\left[{h}_{mask};{h}_{1};{h}_{mask};{h}_{2};\cdots ;{h}_{m};{h}_{mask}\right]$$

To help the decoder identify the positions of the patches, we add new positional embeddings to ${H}_{all}$. Without this, mask tokens would have no information about their location in the image:

$${H}_{dec}={H}_{all}+{E}_{pos}{\prime}$$

Here, ${E}_{pos}{\prime}$ represents the positional encodings for the full set of patches (both masked and unmasked). We use 8 stacked transformer blocks of 16 attention heads as the decoder of NuSPIRe. The final output from the decoder is a sequence of reconstructions $\widehat{X}$, which includes both the originally unmasked patches and the reconstructed versions of the masked patches:

$$\widehat{X}=Decoder\left({H}_{dec}\right)$$

#### Reconstruction and loss function

To reconstruct the image and calculate the loss, we focus specifically on the patches that are masked. We use the mask matrix $M$ to extract the reconstructed patches corresponding to the originally masked patches. The loss is then computed as the mean squared error between the original masked patches and the corresponding reconstructed patches:

$$Loss\left(X,\widehat{X}\right)=\frac{\sum_{i=1}^{N}{M}_{i}{\Vert {X}_{i}-{\widehat{X}}_{i}\Vert }^{2}}{{\sum }_{i=1}^{N}{M}_{i}}$$

This loss drives the model to improve its reconstruction ability.

### Pretraining

We pretrained NuSPIRe using NuCorpus-15 M, a large-scale nuclear image dataset. The images were preprocessed with grayscale conversion, random resized cropping to $112\times 112$ pixels, random horizontal and vertical flipping, and normalization. We split the dataset into 80% training and 20% validation, with a batch size of 800 for training and 2400 for validation. The model, configured a mask ratio of 75%, a patch size of $8\times 8$, and a hidden size of 768, was trained for 70 epochs. We used the AdamW optimizer with a learning rate of 0.0001 and a warm-up phase of 10 epochs. A step scheduler was applied to reduce the learning rate by half every 20 epochs. The training process was distributed across 4 NVIDIA A800 80 GB GPUs using the Distributed Data Parallel strategy and lasted approximately 7 days. The best models were saved based on validation loss.

### Fine-tuning

After the pretraining phase, NuSPIRe can be fine-tuned for downstream tasks. Unlike the pretraining phase where patches are masked, in the fine-tuning phase, no patches are masked. The mask matrix $M$ is set to zero for all elements:

$${M}_{i}=0, \forall i$$

This means all patches are visible during fine-tuning, and the model processes the entire image without any masking. This approach allows the model to utilize full information from the input image based on the entire patch sequence.

The fine-tuning is based on the representation token ${h}_{rep}$, which serves as a comprehensive representation of the input image:

$${h}_{rep}={Encoder}_{\theta }\left(Z\right)[0]$$

Here, ${Encoder}_{\theta }()$ denotes the pretrained encoder of NuSPIRe, with $\theta$ representing its parameters. The representation token ${h}_{rep}$ is then passed into a prediction head, which can be customized based on the specific requirements of the task, typically implemented as either a linear layer or a multi-layer perceptron (MLP).

$$\widehat{Y}={Prediction\_head}_{\phi }\left({h}_{rep}\right)$$

where $\widehat{Y}$ represents the prediction output of model.

We employed both full fine-tuning and partial fine-tuning strategies to adapt the pretrained NuSPIRe for downstream tasks:

1. For full fine-tuning, all parameters of encoder are updated, allowing the model to fully adapt to the new task. The optimization objective is to minimize the specific loss:

$$\theta ,\phi =\mathrm{arg}\underset{\theta ,\phi }{\mathrm{min}}{Loss}{\prime}(Y,{Prediction\_head}_{\phi }\left({Encoder}_{\theta }\left(Z\right)[0]\right))$$

Here, ${Loss}{\prime}()$ represents the loss function of the specific downstream task and $Y$ represents the ground truth of this task.

2. For partial fine-tuning, the encoder parameters are frozen, and only the prediction head is updated. This approach preserves the pretrained features and reduces the risk of overfitting on smaller datasets. The optimization objective focuses on minimizing the task-specific loss by adjusting only the parameters of the prediction head:

$$\phi =\mathrm{arg}\underset{\phi }{\mathrm{min}}{Loss}{\prime}(Y,{Prediction\_head}_{\phi }\left({h}_{rep}\right))$$

In both fine-tuning strategies, appropriate loss functions, optimizers, learning rate schedules, and other hyperparameters are selected based on the specific requirements of the downstream tasks.

### Human NSCLC dataset and dataset preparation

We utilized spatial transcriptomics data from NSCLC tissue sample 5–1 on the CosMx SMI platform for the classification task. We extracted sub-images measuring 20 µm per side from parallel high-resolution DAPI-stained images. Cells were filtered based on gene expression and fluorescence intensity, and subsequently categorized into three major groups according to their annotated cell types. ‘T CD8 naive’, ‘T CD8 memory’, ‘Treg’, ‘T CD4 naive’, ‘T CD4 memory’, ‘NK’, and ‘B-cell’ were grouped as ‘Lymphocytes’; all tumor subtypes were combined into a single ‘Tumor cells’ category; and the remaining cell types were classified as ‘Other cells’. A total of 81,825 cells were included in the analysis, comprising 16,081 tumor cells and 15,105 lymphocytes. Input images were resized to 112 × 112 pixels, followed by data augmentations, including random horizontal and vertical flips and normalization. The dataset was divided into training, validation, and test sets in an 8:1:1 ratio. To mitigate class imbalance, we randomly selected an equal number of cells from each type in the training set for fine-tuning. To evaluate model performance across different dataset sizes, we created multiple subsets with progressively fewer cells per type, repeating the process five times for each subset. Both model selection and performance evaluation were conducted using the same validation and test sets.

### Model comparisons and training strategy for identifying cell types

We compared NuSPIRe to several widely used deep learning models for computer vision, including VGG [49], ConvNeXt [50], ResNet [51], EfficientNet [52], Swin Transformer [53], and ViT [54]. In addition, we introduced two additional methods, namely a masked autoencoder model CA-MAE [55] and a Random Forest classifier trained on classical morphological features. For fair comparison, all deep learning models were trained under three settings, including partial fine-tuning, full fine-tuning, and training from scratch, with their respective pretrained weights used in the fine-tuning settings. We employed the AdamW optimizer with a learning rate that linearly increased to 0.0001 over the first five warm-up epochs. The loss function used was CrossEntropyLoss. For all tasks except NuSPIRe partial fine-tuning, the batch size was set to 300, and the models were trained for 30 epochs. In the case of partial fine-tuning, we extended the training epochs appropriately and adjusted the batch size as needed. Larger datasets used a batch size of 300, while smaller datasets had progressively reduced batch sizes to ensure stable training.

### Performance evaluation metrics for identifying cell types

For performance evaluation, we selected the checkpoint with the lowest loss as the final model for each method. The metrics calculated included Accuracy, F1 Score, Precision, Recall, and AUROC, with the latter four metrics averaged using the ‘macro’ method. Additionally, MCC (Matthews Correlation Coefficient) and Cohen’s Kappa were used to further assess classification performance, particularly in the context of imbalanced datasets.

### Attention rollout for model interpretability

To analyze the interpretability of the NuSPIRe, we applied the Attention Rollout method [20]. This method aggregates attention weights across all transformer layers to provide a cumulative view of how the model attends to different input patches. Specifically, we compute the attention scores at each layer and recursively multiply them through all layers to propagate attention across the network. This allows us to visualize the final attention distribution over the input image, helping to identify the patches that contributed most to the model’s predictions.

### Drug-induced cellular senescence dataset and dataset preparation

The dataset consists of DAPI-stained nuclear images of A549 human lung adenocarcinoma cells, including cells treated with the chemotherapeutic agent etoposide and a control group treated with DMSO [9]. Etoposide-treated cells were used to represent the senescent population, while DMSO-treated cells served as the non-senescent control group. Using CellProfiler [10], nuclei were segmented and extracted from the high-resolution images. Each nuclear image was cropped to a standard size of 50 µm per side, with regions beyond the nuclear boundaries masked to ensure focus on the nuclear features. After filtering for image quality, a total of 30,000 nuclear images were retained for analysis, with an equal distribution of 15,000 senescent and 15,000 non-senescent cells.

### Clustering of Morphological Representations Extracted by NuSPIRe

We applied several commonly used clustering algorithms to the extracted representations, grouping them into two clusters. The Agglomerative Clustering, Spectral Clustering, K-Means, and Gaussian Mixture Method were implemented using the scikit-learn package. The ‘KNN’ and ‘Leiden’ methods were based on a k-Nearest Neighbors graph with k set to 10. The ‘KNN’ method refers to applying spectral clustering on the k-NN graph to achieve neighbor-based clustering, while the ‘Leiden’ method is a graph-based clustering algorithm that controls the number of clusters using the resolution parameter. We then calculated Precision, Accuracy, Recall, and F1 Score to assess the alignment between the clustering labels and the ground truth labels.

### CPJUMP1 dataset

This dataset includes cell images following chemical and genetic perturbations (CRISPR knockout and ORF overexpression). We used DNA images from the primary CPJUMP1 experiment [22], specifically from batch ‘2020_11_04_CPJUMP1’, selecting 10 plates of U2OS cells observed at long time points. For each well of the plates, we randomly selected 4 site images and cropped each nuclear image from the original at 50 µm per side, masking the areas outside the nuclear regions. In total, we obtained 1,423,075 cells, covering 303 compounds, 176 ORF target genes (including ‘none’ targets), and 161 CRISPR target genes (including ‘none’ targets). The median number of cells per perturbation was 1,631.

### MMD between control and perturbation-induced nuclear morphology

MMD (Maximum Mean Discrepancy) is a commonly used statistical method for quantifying the difference between two distributions. In this study, we utilized MMD to measure the morphological changes in cell nuclei induced by different perturbations. The calculation was based on the Radial Basis Function (RBF) kernel, with the Gamma parameter derived from the negative control data to ensure accurate comparisons. Cells subjected to the same perturbation—such as treatment with the same compound, CRISPR or ORF overexpression targeting the same gene—were grouped as a single distribution. We randomly sampled 10% of the negative control group for the analysis. To ensure similar sample sizes across distributions, the number of cells for each perturbation was adjusted through oversampling or downsampling to match the size of the negative control group. MMD was then calculated between each perturbation-induced distribution and its corresponding negative control to evaluate the impact of the perturbation. This approach allowed us to systematically assess differences in nuclear morphology across various perturbation types.

### Identifying distinct significant perturbations

After calculating the MMD for each perturbation type, we analyzed the distribution of MMD values to identify perturbations that caused notable nuclear morphological changes. Outliers were identified using the interquartile range (IQR) method, with perturbations exceeding the threshold of $Q3 + 1.5 \times IQR$ (where $Q3$ represents the third quartile) considered as significant. These outliers were interpreted as distinct significant perturbations, as they indicated a substantial deviation from the baseline nuclear morphology.

### Calculation of cosine similarity across perturbations

To evaluate the similarity between different perturbations, we first computed the mean of the cellular representations across all cells within each perturbation group, generating an overall profile for each treatment. To capture treatment-induced changes, we subtracted the profile of the negative control from each perturbation’s profile, resulting in a corresponding change vector. Cosine similarity was then calculated between these change vectors to quantify the similarity between different perturbations, providing insights into how similarly they affected nuclear morphology.

### Mouse brain tissue dataset and common morphological features calculation

Spatial transcriptomics data were obtained from Formalin-fixed, paraffin-embedded tissue samples of wild-type male mouse brains at three ages: 2.5, 5.7, and 13.4 months, provided by Xenium. DAPI-stained images for each cell were cropped to 15 µm per side and underwent quality screening. After filtering, 53,073, 53,388, and 52,630 cells were retained from the three respective age groups. Data processing and partitioning followed the procedures outlined in the Human NSCLC Dataset and Dataset Preparation section. The common morphological features extracted from the images included nucleus aspect ratio, fluorescence intensity, and nuclear area. The aspect ratio was calculated as the ratio of the smallest bounding rectangle aligned with the nucleus’s longest axis. Fluorescence intensity was computed as the mean pixel value of 16-bit depth images, while nuclear area was derived from dataset annotations. High and low gene expression levels were defined as the top and bottom 5% of expression values across all cells.

### Model comparisons and performance evaluation for predicting gene expression profiles

For model comparisons, the 5.7-month age group data were used with MSELoss as the loss function and the AdamW optimizer (initial learning rate: 0.0001). NuSPIRe was fully fine-tuned, and all other training procedures followed the approach outlined in the Model Comparisons and Training Strategy for Identifying Cell Types section. Gene expression for each cell was predicted using the model with the lowest loss for each comparison. Performance on the test set was evaluated using the following metrics: Pearson Correlation Coefficient (PCC), Spearman Correlation Coefficient (SCC), R^2^, Adjusted R^2^, Concordance Correlation Coefficient (CCC), Explained Variance (EV), Root Mean Squared Error (RMSE), Mean Squared Error (MSE), Mean Absolute Error (MAE), and Quantile Loss (QL). For gene-wise evaluation, PCC was computed between the predicted and ground truth expression values for each gene.

### Tertiary Lymphoid Structure (TLS) dataset and data preparation

The dataset was derived from mIHC imaging of 64 patients diagnosed with ESCC. Each mIHC image was stained with DAPI, CD3, and CD20 to facilitate the identification of TLS. The dataset was divided into a training and validation set consisting of 38 image slices and a test set comprising 26 image slices. The training and validation set was further split at a ratio of 9:1. To address data imbalance, the training set was structured to maintain a 1:10 ratio of positive (TLS) to negative (non-TLS) samples, with negative samples randomly selected to be 10 times the number of positive samples.

The whole slide images were segmented into smaller patches of 256 × 256 pixels using a sliding window approach. For the training set, the sliding window step size was set to 256 pixels. In the test set, to achieve more detailed predictions, patches were generated with a step size of either 256 pixels (equal to the patch side length) or 128 pixels (half of the patch side length). Each patch was labeled based on the quantity and intensity of CD3 and CD20 cells within its boundaries to determine whether it represented a TLS region. Both the cell count and the staining intensity were considered for precise classification. Patches that were entirely black (i.e., lacking any DAPI, CD3, or CD20 staining) were discarded to maintain dataset quality by removing irrelevant data.

The dataset used for prediction comprised spatial transcriptomics data from NSCLC tissue sample 5–3, obtained through the CosMx SMI platform. For patch generation during segmentation, a sliding window approach was employed with a step size set to one-quarter of the patch side length. Each patch had dimensions of 912 × 912 pixels, chosen to accommodate the size of the images. The cell type annotations for the dataset were provided by the official CosMx Dataset website.

### Training strategy for identifying tertiary lymphoid structures

The training strategy utilized Focal Loss as the loss function, with default hyperparameters to address class imbalance by focusing more on hard-to-classify examples. The AdamW optimizer was employed, with a learning rate that linearly increased to 0.00001 over the first five warm-up epochs to ensure stable convergence. The batch size for training was set at 200, and training was conducted over 20 epochs. The model corresponding to the epoch with the lowest loss was selected as the final model to ensure optimal performance.

### Calculation of TLS Scores and T Cells & B cells captured proportion

The TLS Scores were calculated by directly inputting the data into our fine-tuned model, with the resulting predicted probabilities representing the TLS Scores. For calculating the T Cells & B Cells Captured Proportion, three different sequencing methods were employed. In the NuSPIRe-Guided Sequence, the TLS Scores were sorted in descending order and then cumulatively summed. The Scanning Sequence involved accumulating the values based on the sequential order of the FOV numbers. Lastly, the Random Sequence method involved randomly shuffling the FOVs 10 times and then calculating the mean of the resulting values.

### Immune infiltration dataset and model training

Spatial transcriptomics data from NSCLC tissue samples were obtained using the CosMx SMI platform. Preprocessing, including patch generation and cell type annotation, followed the same procedure described in the Tertiary Lymphoid Structure (TLS) Dataset section. For each image patch, the numbers of T and B cells were quantified to compute infiltration scores. The dataset was divided into training, validation, and test sets in an 8:1:1 ratio. The model was trained to predict infiltration scores using the AdamW optimizer with a learning rate linearly increased to 0.0001 over the first three warm-up epochs and optimized with mean squared error (MSE) loss for 20 epochs with a batch size of 256. The model achieving the lowest validation loss was selected as the final version for inference.

## Supplementary Information

Additional file 1: Figs. S1 to S14, providing additional supporting results and validations for NuSPIRe, including benchmarking, unsupervised analyses, morphology-gene expression associations, and downstream spatial omics applications.

Additional file 2: Tables S1 to S3, summarizing supplementary statistics and key datasets used in the study, including perturbation results and dataset overviews.

## Data Availability

The NuSPIRe source code is publicly available under the MIT License via GitHub [56] and the Hugging Face Model Hub [57], and has also been archived on Zenodo [58]. NuCorpus-15 M is publicly available on Zenodo as a series of dataset records (Parts 1–3) [59–61]. All datasets used in this study are publicly available (see Additional file 2: Table S3 for details).
