# Supplementary material for: Self-supervised pretraining with NuSPIRe unlocks nuclear morphology-driven insights in spatial omics
Source: Genome Biol. 2026 Feb 3;27:71. doi: 10.1186/s13059-026-03987-2 (PMC12958554; doi:10.1186/s13059-026-03987-2)
Supplement: Supplementary file 1 — Additional file 1: Figs. S1 to S14, providing additional supporting results and validations for NuSPIRe, including benchmarking, unsupervised analyses, morphology-gene expression associations, and downstream spatial omics applications. [file 13059_2026_3987_MOESM1_ESM.docx]

**Supplementary Figures**

**
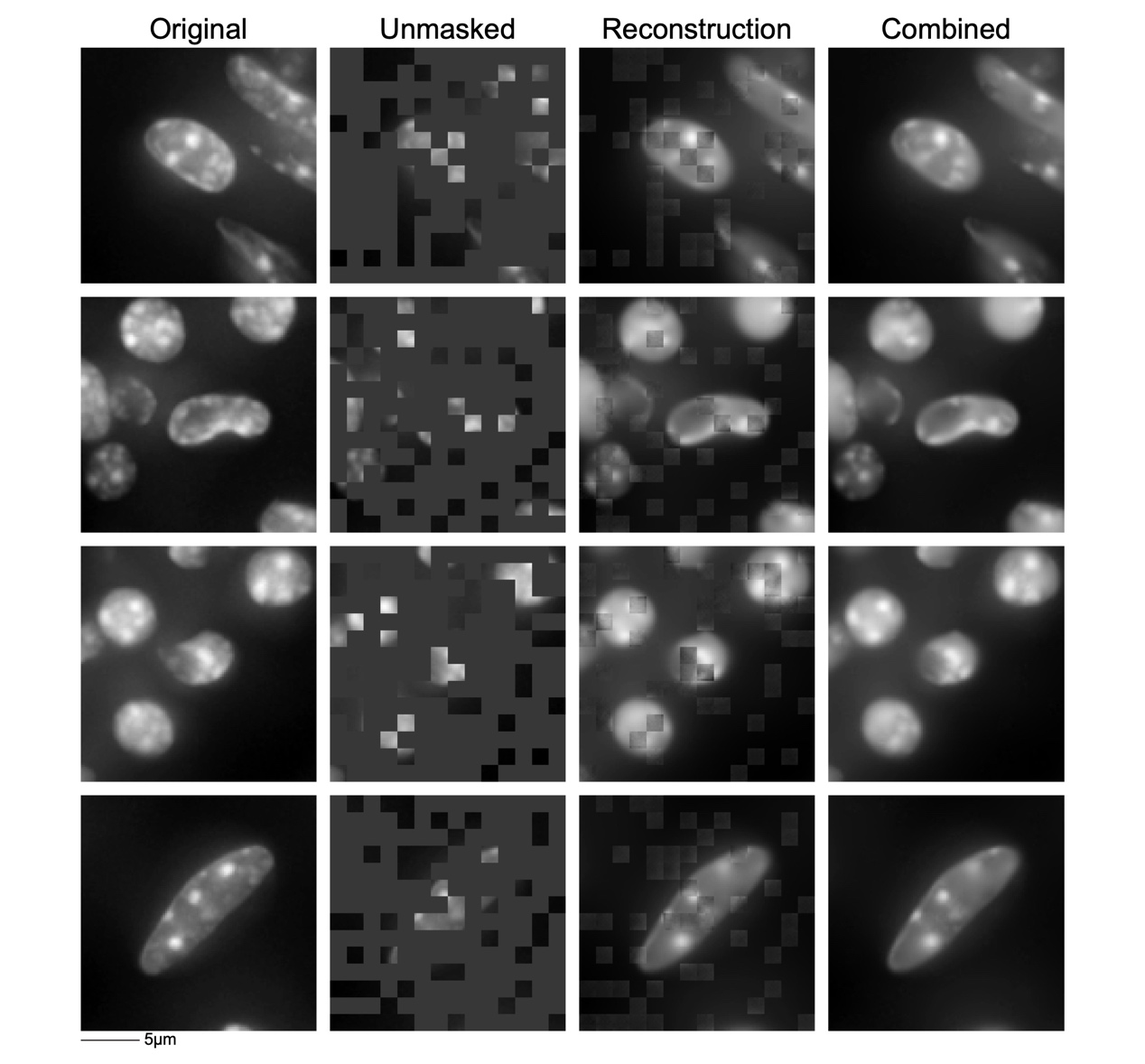
**

**Fig S1: Reconstruction Performance of NuSPIRe.**

The figure presents the original input image, the unmasked portions, the reconstructed output, and the combined output, illustrating the model's effectiveness in reconstruction.

**
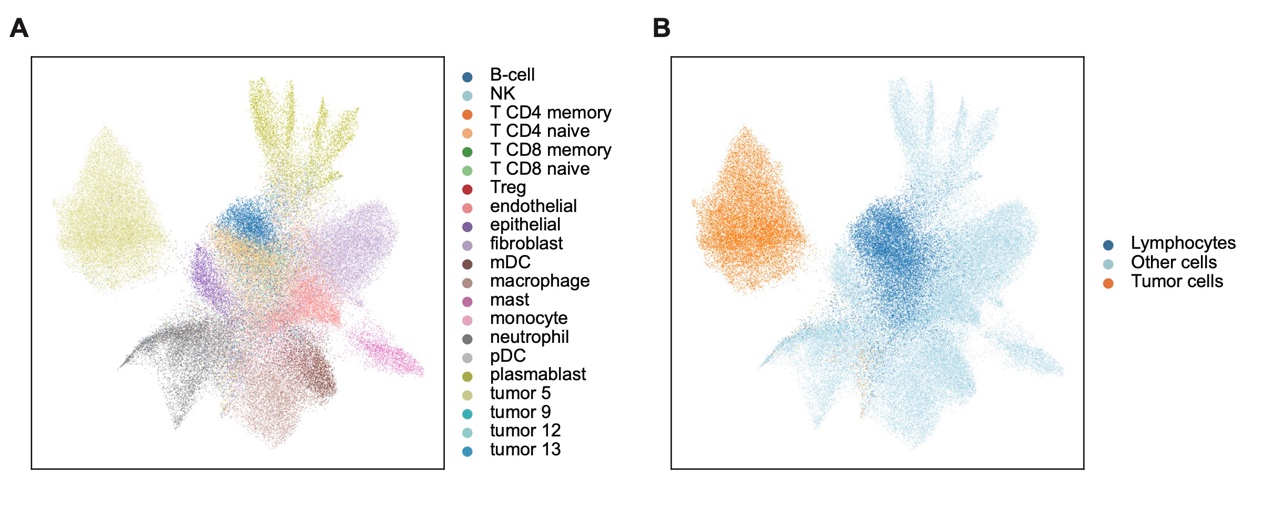
**

**Fig S2: Cell Type Classification of NSCLC Dataset.**

(A) UMAP projection of cells colored by their original classifications based on gene expression profiles. (B) UMAP projection of the same cells reclassified into lymphocytes, tumor cells, and other cell types.


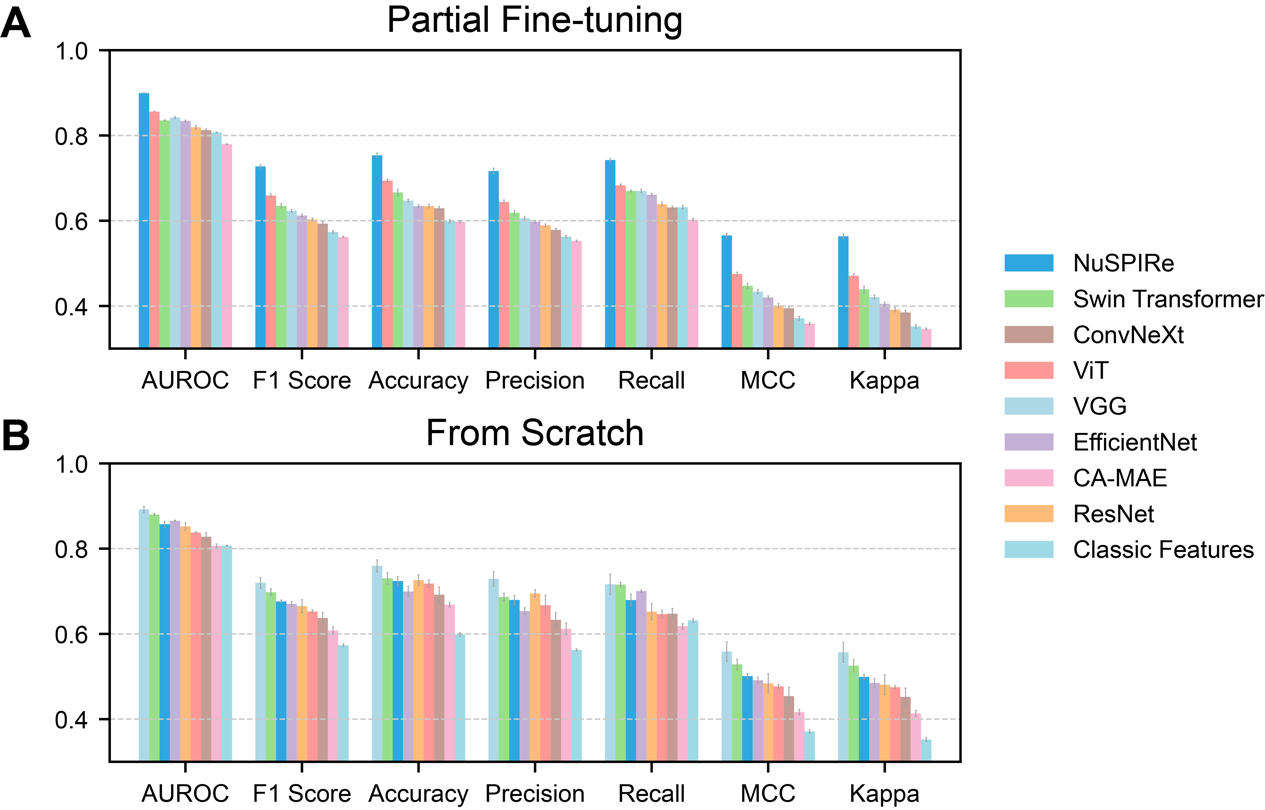


**Fig S3: Benchmarking across training settings and baseline methods.**

(A) Partial fine-tuning (frozen encoder with a trained prediction head). (B) Training from scratch. Performance is summarized using seven metrics (AUROC, F1 score, accuracy, precision, recall, MCC, and Cohen’s κ). The benchmark compares NuSPIRe with Swin Transformer, ConvNeXt, ViT, VGG, EfficientNet, and ResNet. The masked-autoencoder baseline method CA-MAE and a random forest trained on classic features are also included.


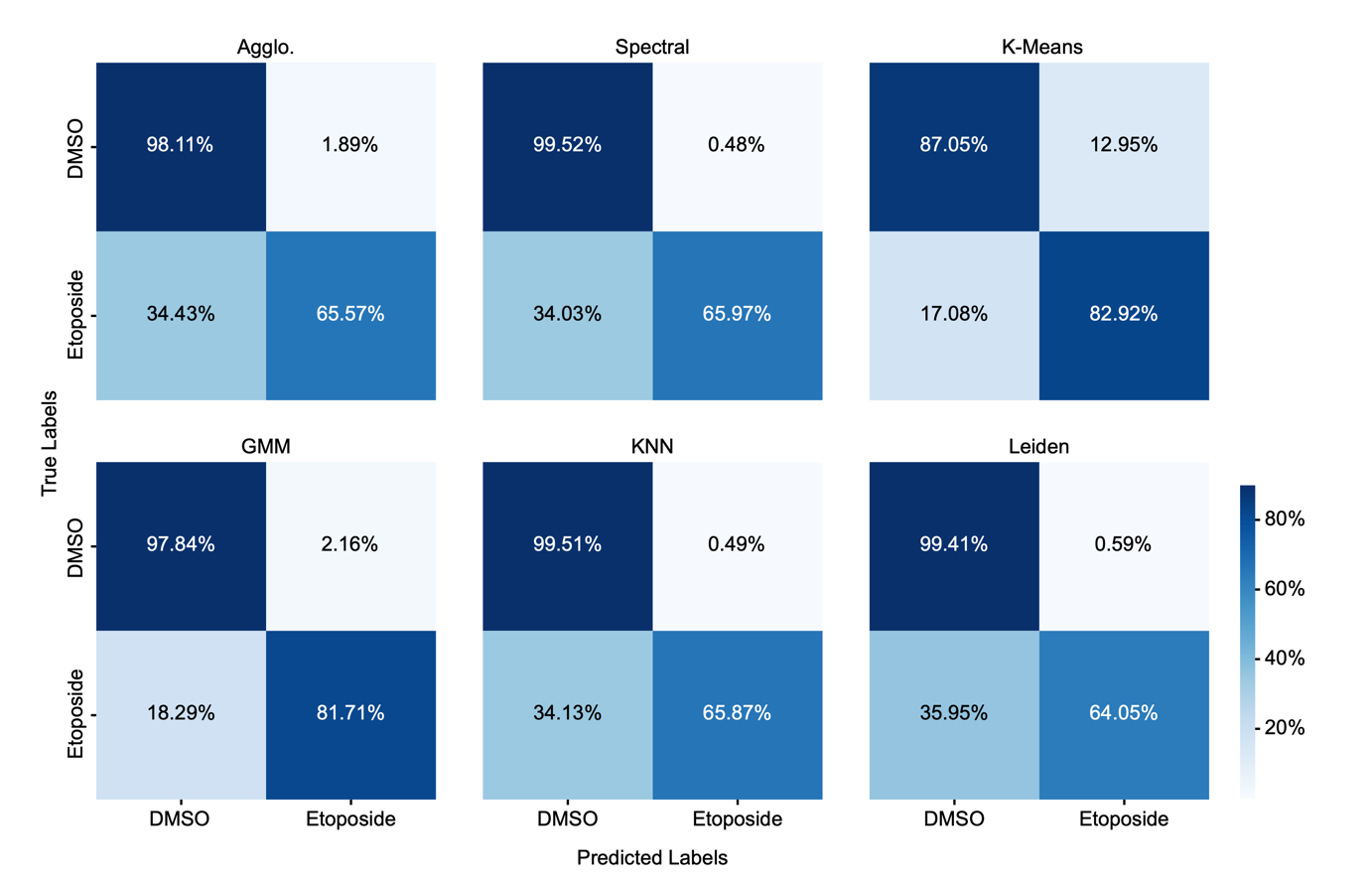


**Fig S4: Clustering Performance of Various Unsupervised Methods on NuSPIRe Representations.**

Confusion matrices for six clustering methods, Agglomerative clustering (Agglo.), Spectral Clustering, K-Means, Gaussian Mixture Model (GMM), k-Nearest Neighbors (KNN) and Leiden, classifying DMSO- and etoposide-treated cells based on representations extracted by NuSPIRe.


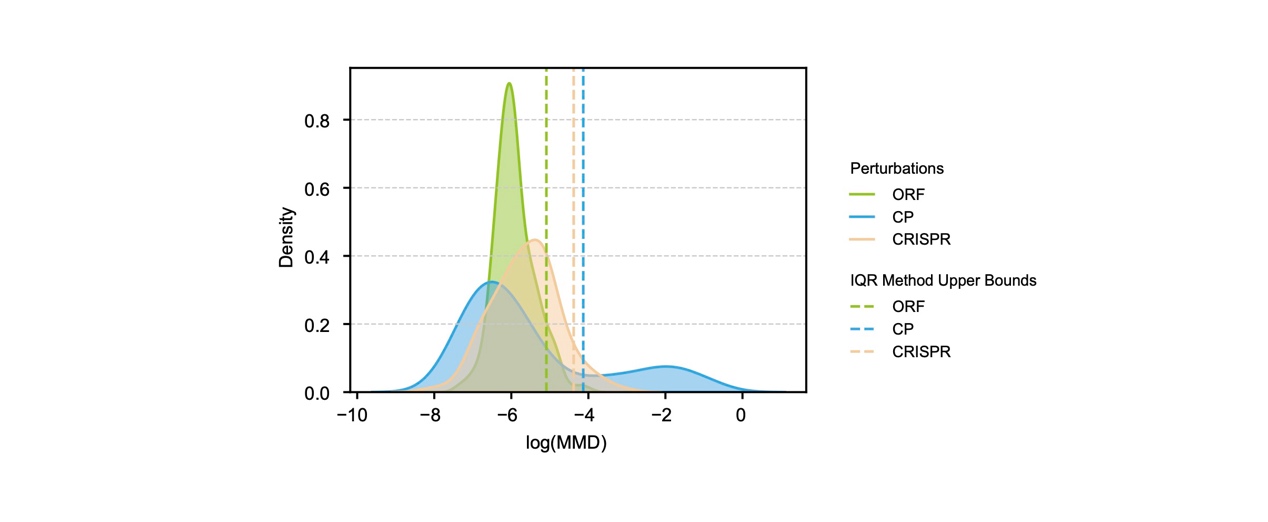


**Fig S5: Differences in MMD Distribution Between Different Types of Perturbations.**

Distribution of MMD between perturbations (ORF, CRISPR, CP) and the DMSO control group. The x-axis represents log-transformed MMD values, and the y-axis represents density. Upper bounds are calculated using the interquartile range (IQR) method (1.5 IQR + Q3), with values exceeding the upper bounds considered significant perturbation.


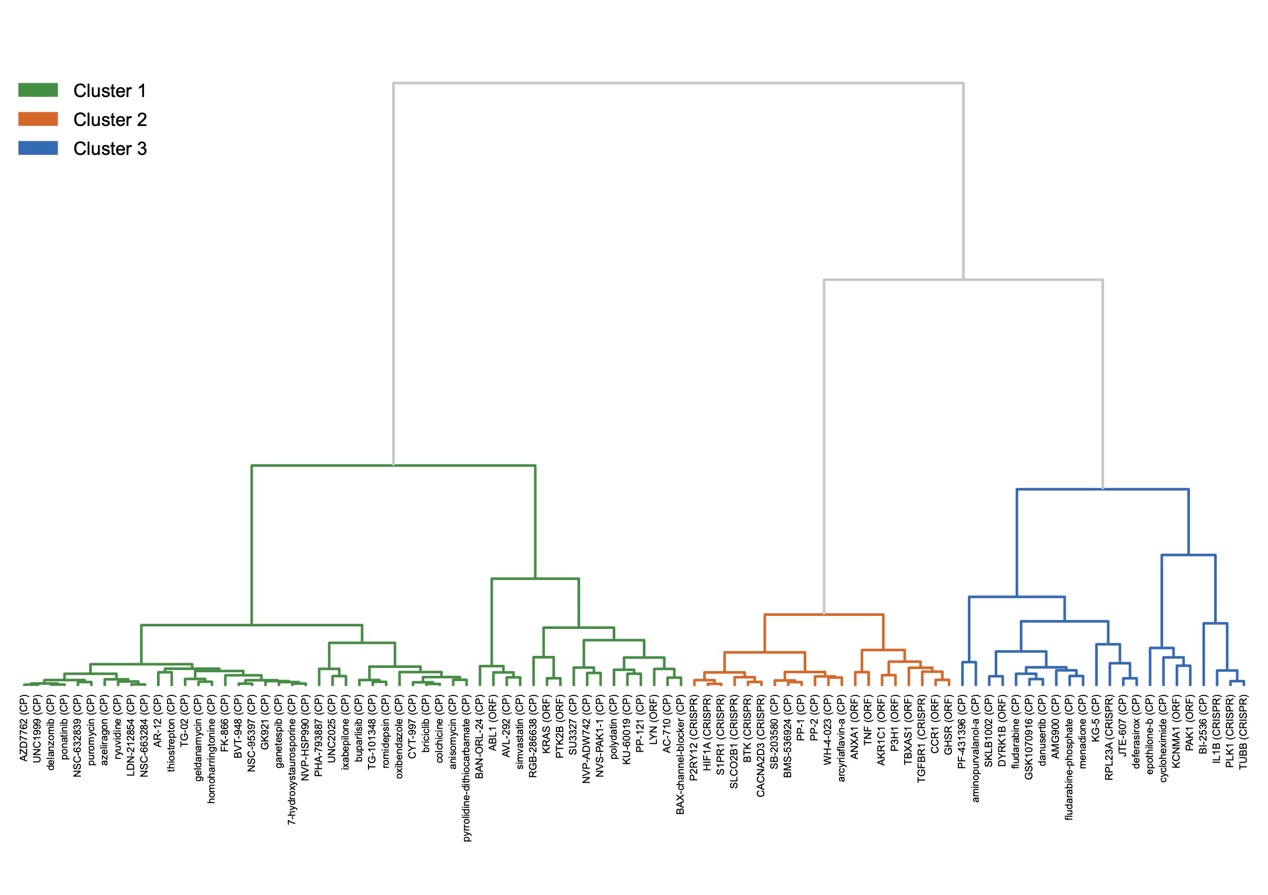


**Fig S6: Classification of Distinct Significant Perturbations.**

Hierarchical clustering of distinct significant perturbation vectors based on pairwise cosine similarity is presented. Each terminal node in the dendrogram represents a perturbation, and the clustering reveals three distinct clusters.


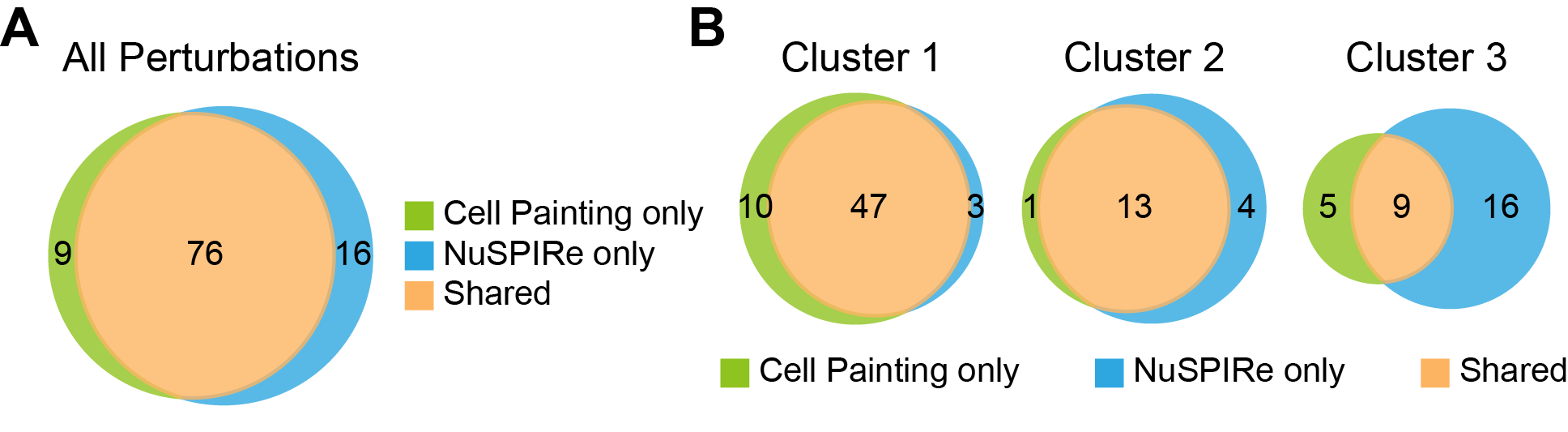


**Fig S7: Comparison of perturbation detection between NuSPIRe and Cell Painting features in the CPJUMP1 dataset.**

(A) Overlap of significant perturbations identified by NuSPIRe with nuclear morphology features only and by classical features from all Cell Painting channels, using the same MMD-based annotation-free detection pipeline. (B) Correspondence of morphological clusters obtained by hierarchical clustering of perturbation vectors derived from NuSPIRe and Cell Painting. Clusters 1-3 show consistent grouping of biologically related perturbations.


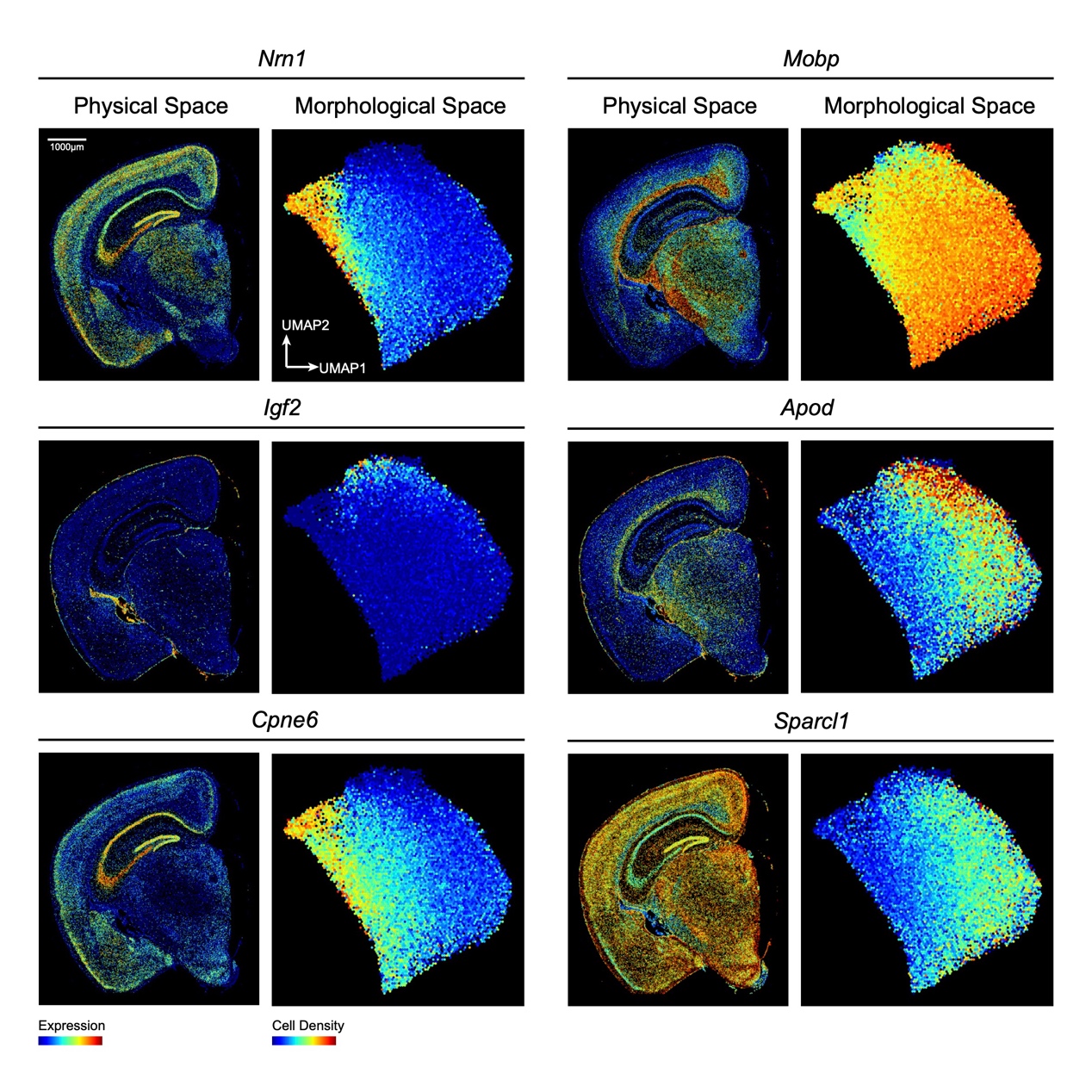


**Fig S8: Spatial Expression Patterns and Morphological Distribution of Genes.**

Genes (*Nrn1, Mobp, Igf2, Apod, Cpne6, Sparcl1*) in the mouse brain at 5.7 months are presented. For each gene, spatial expression maps show cells colored according to gene expression levels, while UMAP projections in morphological space depict cells colored based on cell density.


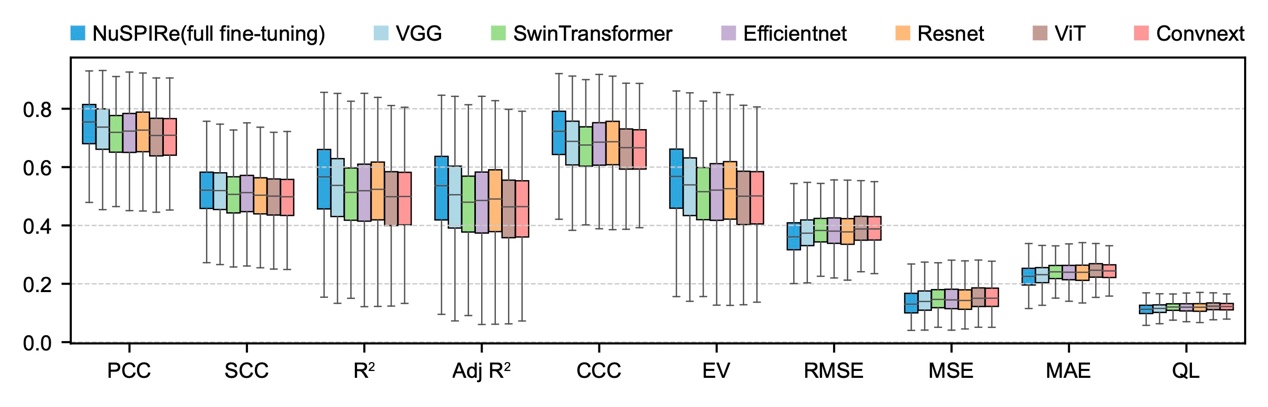


**Fig S9: Comparison of Model Performance in Predicting Gene Expression in the Mouse Brain at 5.7-months.**

Each box represents the distribution of prediction performance across all cells for the 347 genes included in the spatial transcriptomics panel, evaluated for each model, including NuSPIRe (full fine-tuning), VGG, SwinTransformer, EfficientNet, ResNet, ViT, and ConvNeXt. Performance is evaluated using several metrics: Pearson Correlation Coefficient (PCC), Spearman Correlation Coefficient (SCC), R², Adjusted R², Concordance Correlation Coefficient (CCC), Explained Variance (EV), Root Mean Squared Error (RMSE), Mean Squared Error (MSE), Mean Absolute Error (MAE), and Quantile Loss (QL).


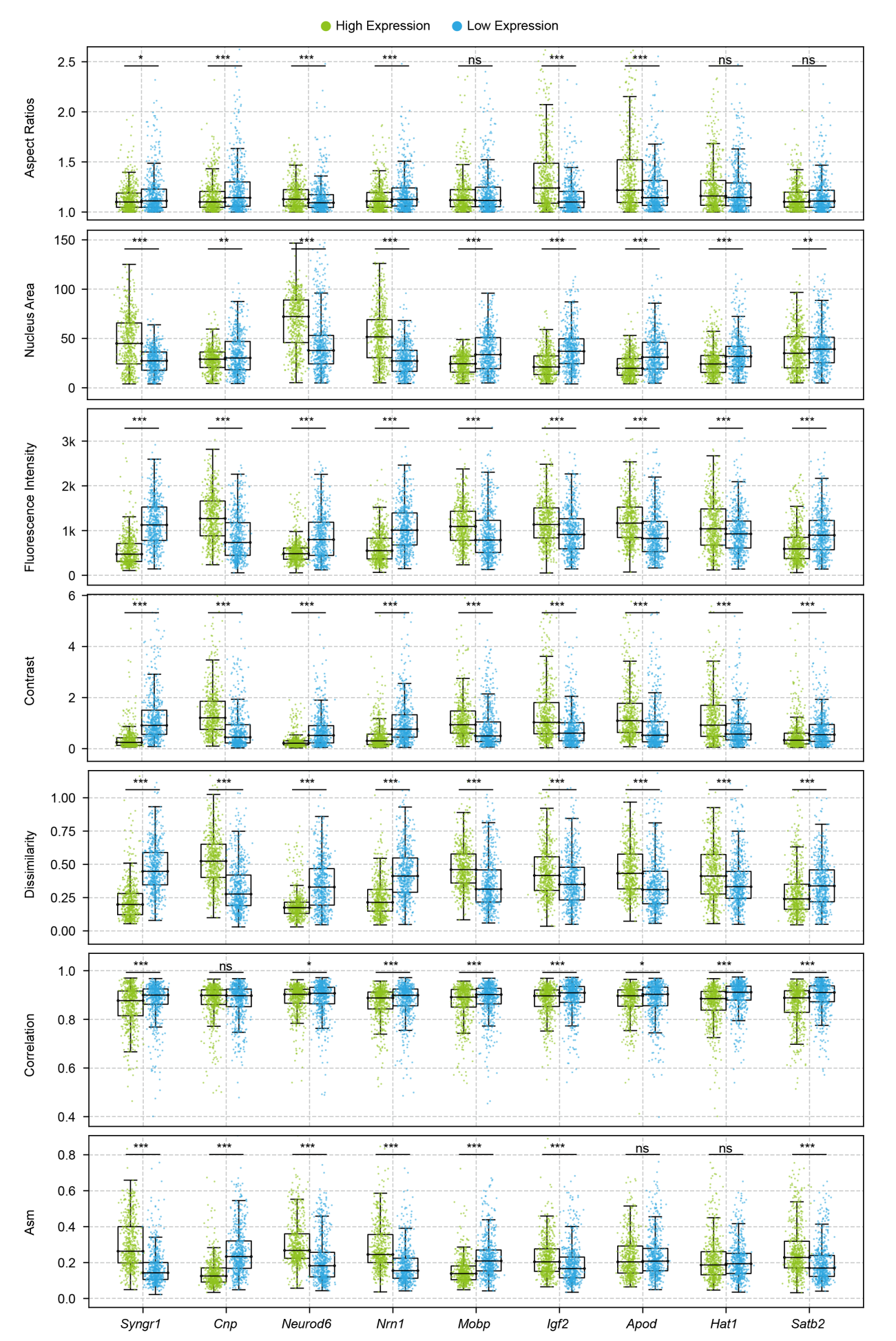


**Fig S10: Distributional Differences in Common Morphological Features Between High and Low Expression Groups.**

Boxplots illustrate the differences in aspect ratio, nuclear area, fluorescence intensity, and texture features (contrast, dissimilarity, correlation, and angular second moment (ASM)) between high and low expression groups for genes *Syngr1, Cnp, Neurod6, Nrn1, Mobp, Igf2, Apod, Hat1,* and *Satb2*. Significant differences are indicated by asterisks, while non-significant differences are denoted as “ns”.


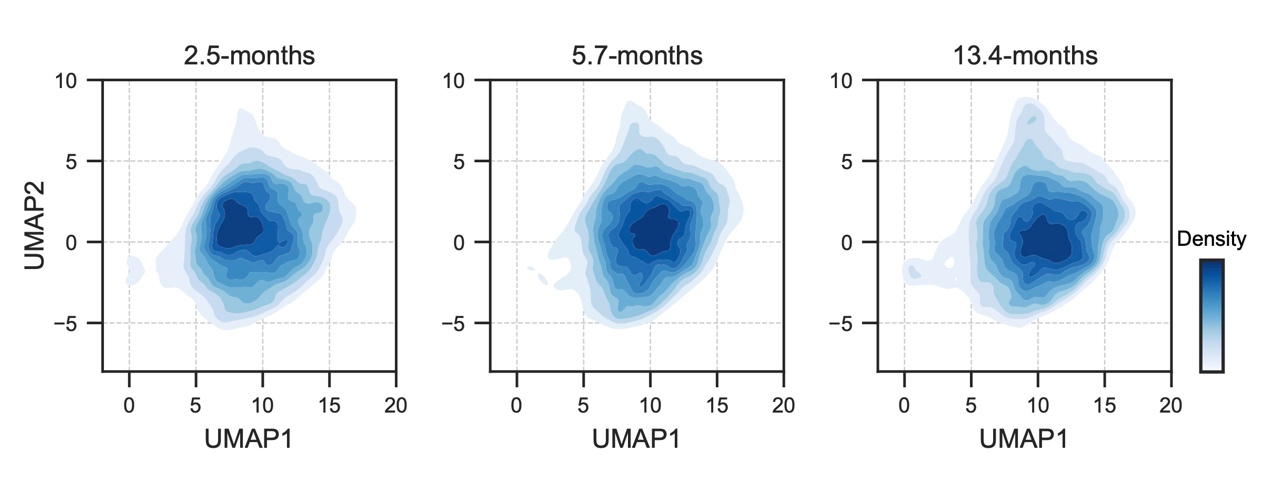


**Fig S11: Cellular Density Variation in the Morphological Space Across Different Developmental Stages.**

UMAP projections illustrating cellular density distribution at three stages: 2.5-months, 5.7-months, and 13.4-months. Each plot visualizes the distribution of cell densities within the corresponding morphological space, with darker regions representing higher cellular density areas.


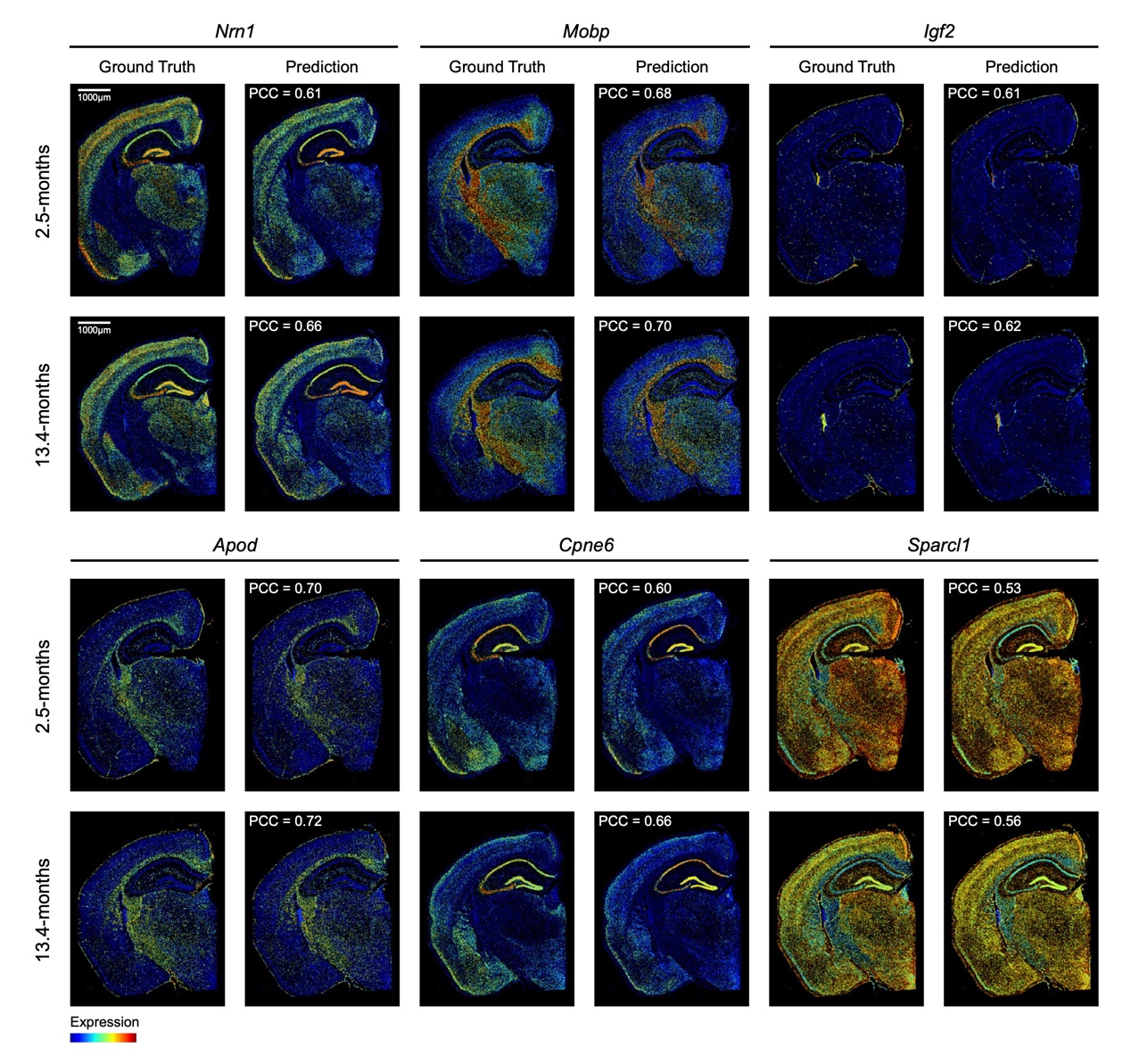


**Fig S12: Gene Expression Prediction Performance at Different Stages in the Mouse Brain.**

Gene expression maps for *Nrn1, Mobp, Igf2, Apod, Cpne6*, and *Sparcl1* are presented at 2.5 and 13.4 months. Pearson correlation coefficients (PCC) between predicted and actual expression levels are provided for each gene at both time points.


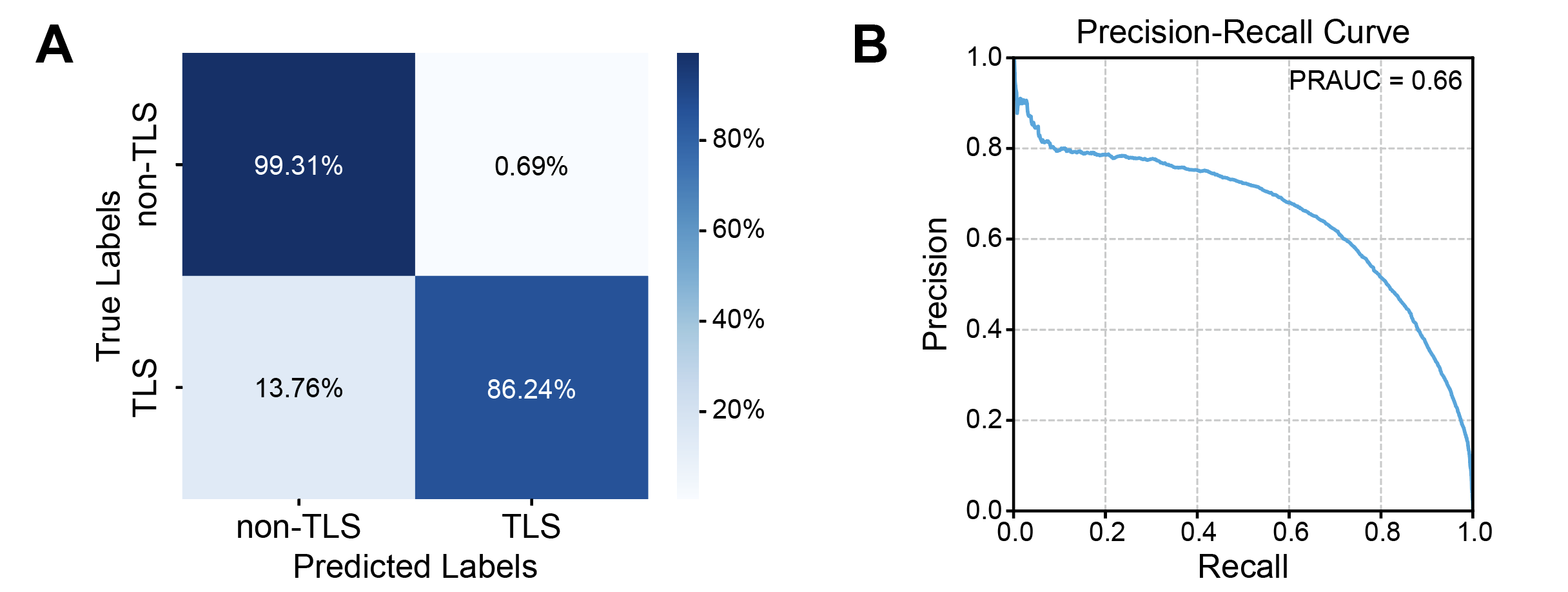


**Fig S13: Model Performance after fine-tuning on the esophageal cancer dataset.**

(A) Confusion matrix on the esophageal cancer test dataset, calculated as an aggregate across different slices, with the horizontal axis indicating predicted classes and the vertical axis indicating true classes. Values are expressed as percentages. (B) Precision-recall curve for TLS region identification using the NuSPIRe model, with a precision-recall area under the curve (PRAUC) of 0.66.


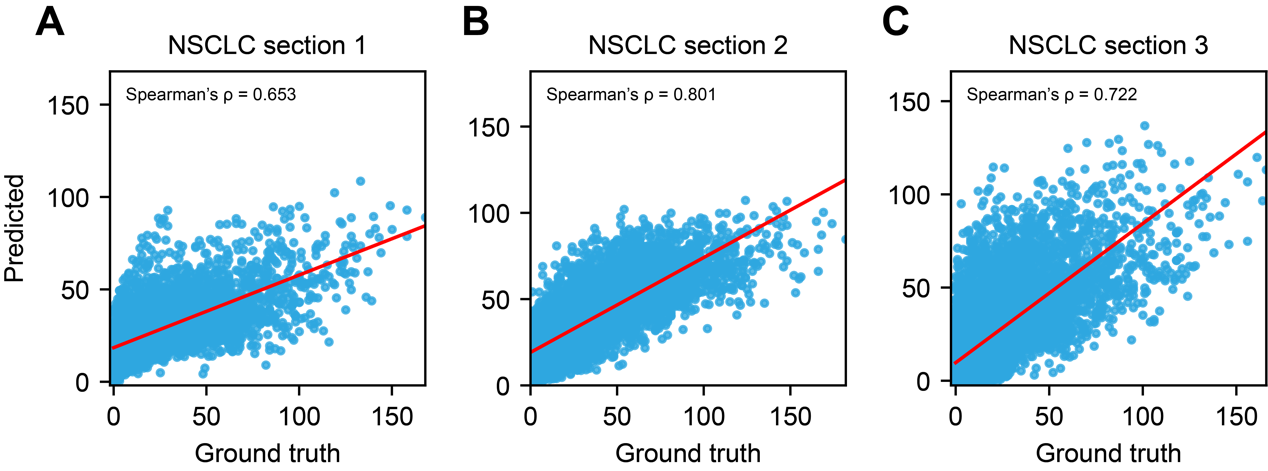


**Fig S14: Prediction of immune infiltration levels from DAPI using NuSPIRe.**

(A–C) Scatter plots showing the correlations between NuSPIRe-predicted immune infiltration scores and the ground-truth B and T cell counts for three independent NSCLC sections. Each dot represents a DAPI image patch. Red lines denote linear regression fits. Spearman’s ρ values quantify the correlation strength in each section.
